# Design, Synthesis, and Biological Evaluation of Boron-Containing Macrocyclic Polyamines and Their Zinc(II) Complexes for Boron Neutron Capture Therapy

**DOI:** 10.1021/acs.jmedchem.1c00445

**Published:** 2021-06-02

**Authors:** Hiroki Ueda, Minoru Suzuki, Reiko Kuroda, Tomohiro Tanaka, Shin Aoki

**Affiliations:** † Faculty of Pharmaceutical Sciences, Tokyo University of Science, 2641 Yamazaki, Noda, Chiba 278-8510, Japan; ‡ Institute for Integrated Radiation and Nuclear Science, Kyoto University, 2-Asashiro-nishi, Kumatori, Osaka 590-0494, Japan; § Research Institute for Science and Technology, Tokyo University of Science, 2641 Yamazaki, Noda, Chiba 278-8510, Japan; ∥ Research Institute for Biomedical Sciences, Tokyo University of Science, 2641 Yamazaki, Noda, Chiba 278-8510, Japan

## Abstract

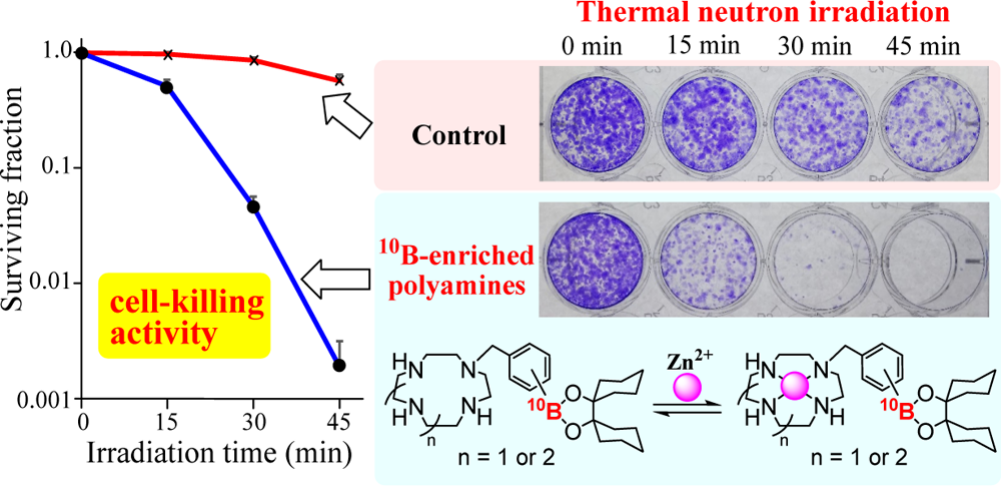

Boron neutron capture therapy (BNCT) is a binary therapeutic method for cancer treatment based on the use of a combination of a cancer-specific drug containing boron-10 (^10^B) and thermal neutron irradiation. For successful BNCT, ^10^B-containing molecules need to accumulate specifically in cancer cells, because destructive effect of the generated heavy particles is limited basically to boron-containing cells. Herein, we report on the design and synthesis of boron compounds that are functionalized with 9-, 12-, and 15-membered macrocyclic polyamines and their Zn^2+^ complexes. Their cytotoxicity, intracellular uptake activity into cancer cells and normal cells, and BNCT effect are also reported. The experimental data suggest that mono- and/or diprotonated forms of metal-free [12]aneN_4_- and [15]aneN_5_-type ligands are uptaken into cancer cells, and their complexes with intracellular metals such as Zn^2+^ would induce cell death upon thermal neutron irradiation, possibly via interactions with DNA.

## Introduction

Boron neutron capture therapy (BNCT) is a potential radiotherapy based on the nuclear reaction between boron-10 (^10^B) atoms and thermal neutrons (^1^n). The neutron capture reaction [^10^B(n, α)^7^Li] generates high linear energy transfer (LET) α particles and lithium ions that have destructive effects and short path lengths in the 5–9 μm range. Therefore, it is expected that cancer cells containing ^10^B species would be selectively destroyed with minimal effects on healthy tissues.^1^

**Scheme 1:**
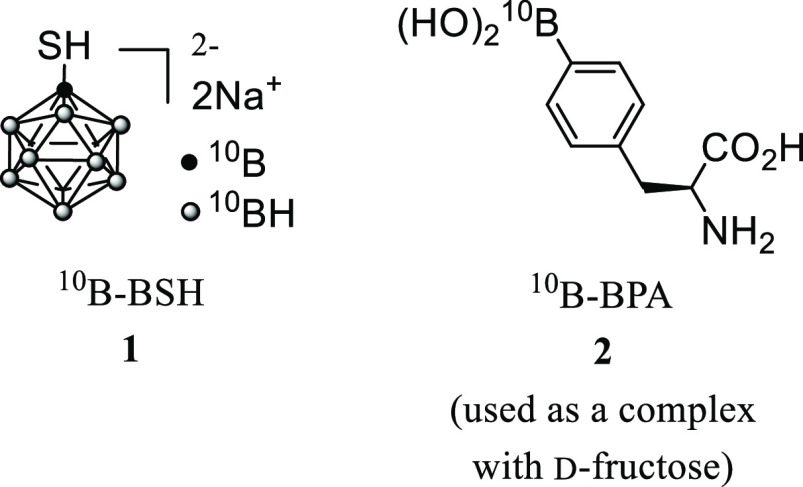
Structures of Representative BNCT Agents

For successful BNCT, a high level of accumulation and selective delivery of ^10^B into cancer cells are required. The design of effective BNCT agents requires the following criteria: (1) low systemic toxicity and higher uptake in tumor tissue than in normal tissue [tumor to blood (T/B) ratios should be greater than 3]; (2) ^10^B must be retained in the tumor tissue but also be rapidly cleared from blood and normal tissues; and (3) the concentration of boron inside or near tumor cells must be ≥10^9 10^B atoms/cell (20–35 μg/gram of tumor tissue).^2^ In this context, only two compounds, sodium mercaptoborate (BSH) **1**(3) and l-4-boronophenylalanine (BPA) **2**(4) (used as a complex with d-fructose) have been used for the clinical treatment of cancers such as malignant glioma, malignant melanoma, and recurrent head and neck cancer, which are not enough for treatment of multiple tumor types (Scheme 1).^5^

To date, numerous boron-containing analogues including amino acids,^6^ biochemical precursors of nucleic acids,^7^ carbohydrates,^8^ amines,^9^ porphyrins,^10^ peptides,^11^ liposomes,^12^ and monoclonal antibodies have been developed.^13^ However, most of them do not satisfy the above criteria for clinical applications. Therefore, more potent boron agents are highly required in order to improve the therapeutic effect and to apply to various tumor types such as breast, lung, and pancreatic cancer.

For the aforementioned purpose, we previously reported on the design and synthesis of sulfoquinovosyl acyl glycerol (SQAG) derivatives and 2-boryl-1,2-dideoxy-d-glucose derivatives, which were possibly transferred into cancer cells through the glucose transporter 1 (GLUT1),^14,15^ because large amounts of d-glucose are consumed by anaerobic glycolysis during the rapid proliferation of cancer cells, which is known as the Warburg effect.^16^ However, their effect on BNCT was not satisfying, despite the moderate intracellular uptake of these agents.

**Scheme 2:**
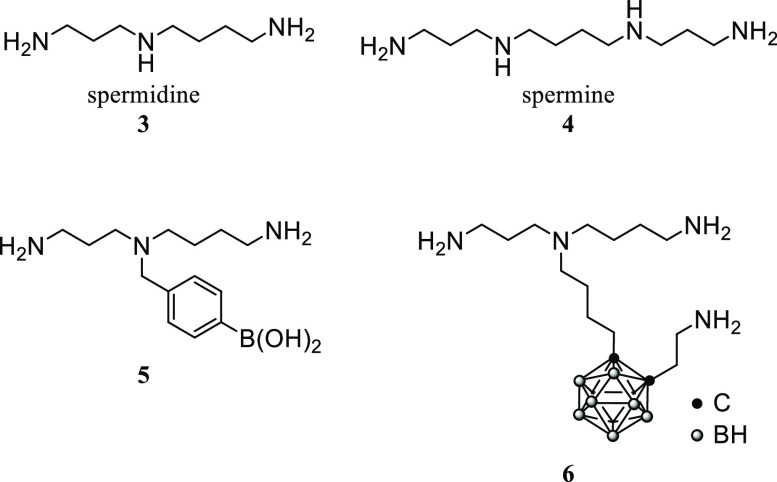
Structures of Polyamines and Boron-Containing Spermidine Derivatives **5** and **6**

It is also known that polyamines including spermidine **3** and spermine **4** are essential for numerous cellular functions such as DNA replication and protein synthesis.^17^ The increase in polyamine concentrations in cancer cells is associated with the activation of cell proliferation and regulated by the promoted polyamine transport system (PTS) and biosynthesis.^18^ Therefore, polyamine derivatives could serve as potentially useful scaffolds for the delivery of boron-containing drugs into cancer cells, as represented by the spermidine derivatives **5** and **6** (Scheme 2).^9,19^ To the best of our knowledge, however, the use of these derivatives in BNCT has not been reported.

**Scheme 3:**
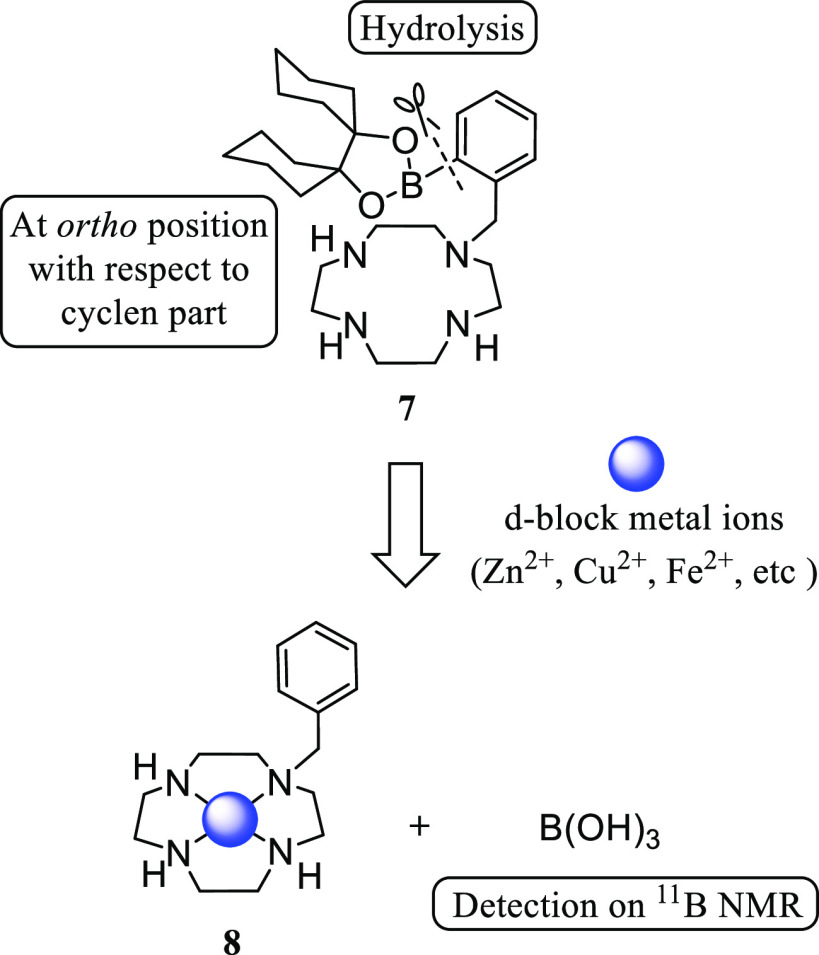
Hydrolytic Cleavage of C–B Bond of **7**

We previously reported on the design and synthesis of phenylboronic acid-pendant cyclen (1,4,7,10-tetraazacyclododecane, [12]aneN_4_) **7** for the sensing of metal cations such as zinc (Zn^2+^), iron (Fe^2+^), copper (Cu^2+^), and cobalt (Co^2+^) (Scheme 3).^20^ It was found that the carbon–boron bond at the *o*-position of the (2-boronophenyl)methyl side chain in **7** is hydrolyzed upon complexation with these metal ions to give **8**, resulting in a shift of the ^11^B NMR signal from ca. 30 ppm to ca. 20 ppm, which corresponds to B(OH)_3_. In addition, we also found that **7** was efficiently transferred into cancer cell lines (Jurkat, A549 and HeLa S3 cells).^15,20^ In subsequent studies, the decomposition of *ortho*-carborane-polyamine conjugates upon metal complexation was discovered and applied to the magnetic resonance imaging (MRI) of Cu^2+^ in solutions.^21^

**Scheme 4:**
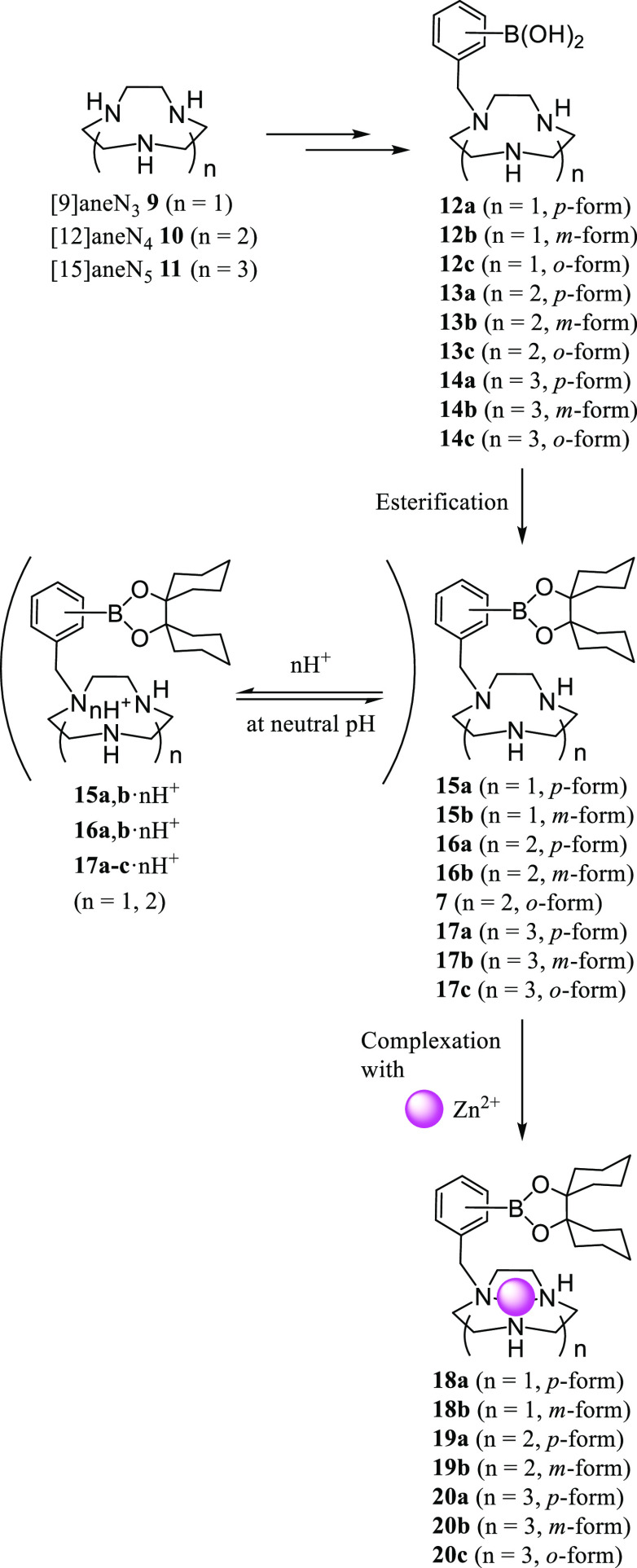
Structures of Macrocyclic Polyamine Derivatives and Their Zn^2+^ Complexes Synthesized in This Work

The aforementioned background and the high intracellular uptake of **7** in cancer cells prompted us to examine the development of boron carriers equipped with macrocyclic polyamine scaffolds such as [9]aneN_3_ (1,4,7-triazacyclononane) **9**, [12]aneN_4_ (cyclen) **10**, and [15]aneN_5_ (1,4,7,10,13-pentaazacyclopentadecane) **11** (Scheme 4). In this work, we designed and synthesized the phenylboronic acid-pendant macrocyclic polyamines **12**–**14**, their corresponding boronic acid ester analogues **15**–**17**, and Zn^2+^ complexes **18**–**20**. It was expected that the cationic charge of **15**–**17** due to the protonation of macrocyclic polyamine groups (**15**–**17**·*n*H^+^, *n* = 1 or 2) would facilitate their intracellular uptake.^18,22,23^ We hypothesized that the protonated form of these boron–polyamine conjugates (**15**–**17**) would be restricted to mono- or dicationic forms (*n* = 1, 2) (**15a**,**b**·*n*H^+^, **16a**,**b**·*n*H^+^, and **17a**–**c**·*n*H^+^ forms in Scheme 4) due to the deprotonation constants of the macrocyclic polyamines, **9**,^24^**10**,^25^ and **11**,^26^ as described below.

**Scheme 5:**
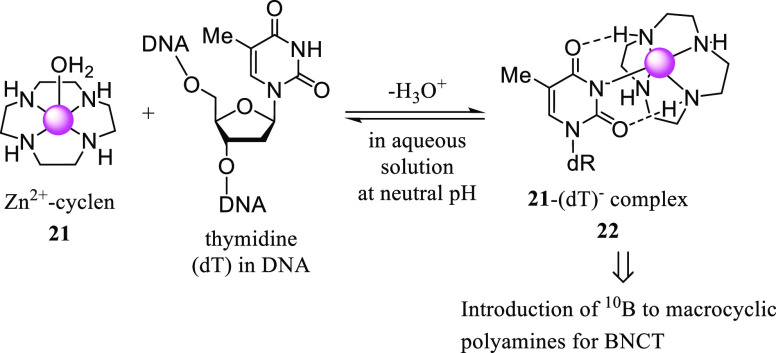
Complexation of Zn^2+^–Cyclen **21** with the Deprotonated dT^–^ in Aqueous Solution at Neutral pH

It is also well known that macrocyclic polyamines form stable complexes with intracellular metal ions such as Zn^2+^, Cu^2+^, and Ni^2+^ in aqueous solutions at physiological pH (Scheme 4),^27−29^ and these complexes are much more stable than Zn^2+^ complexes of linear polyamines such as spermidine **3** and spermine **4**. In addition, it was reported that the cytotoxicity of macrocyclic polyamines is reduced by the complexation with Zn^2+^.^30^ It is well established that Zn^2+^–cyclen complexes such as **21** bind to thymidines (dT) in DNA to form stable complexes **22** through the coordination bonding between the deprotonated imide moiety of dT (dT^–^) and Zn^2+^ in aqueous solution at neutral pH (Scheme 5).^31^ Therefore, we expected that the neutron irradiation of **18**–**20** when located in close proximity to DNA would effectively induce DNA damage. In this study, we report on the cytotoxicity and intracellular uptake activity of **12**–**17** and the corresponding Zn^2+^ complexes **18**–**20** in several cancer cell lines. These agents were first prepared as ligands containing boron in a natural abundance ratio (^10^B/^11^B = 19.9/80.1). After the biological assessment of these ^10^B/^11^B agents, three promising compounds were chosen among them and the corresponding ^10^B-enriched compounds and their Zn^2+^ complexes were synthesized and used in BNCT experiments.

## Results and Discussion

### Synthesis of Boron-Containing Macrocyclic Polyamine Derivatives and the X-ray Single Crystal Structure Analysis of **19a**

**Scheme 6:**
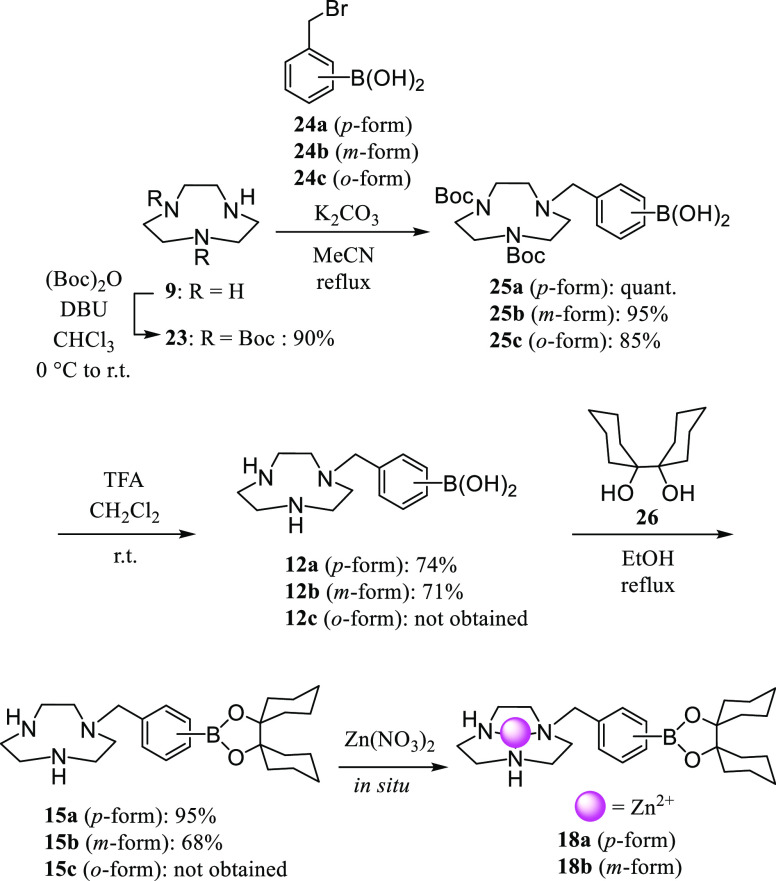
Synthesis of **12a**,**b**, **15a**,**b**, and **18a**,**b**

**Scheme 7:**
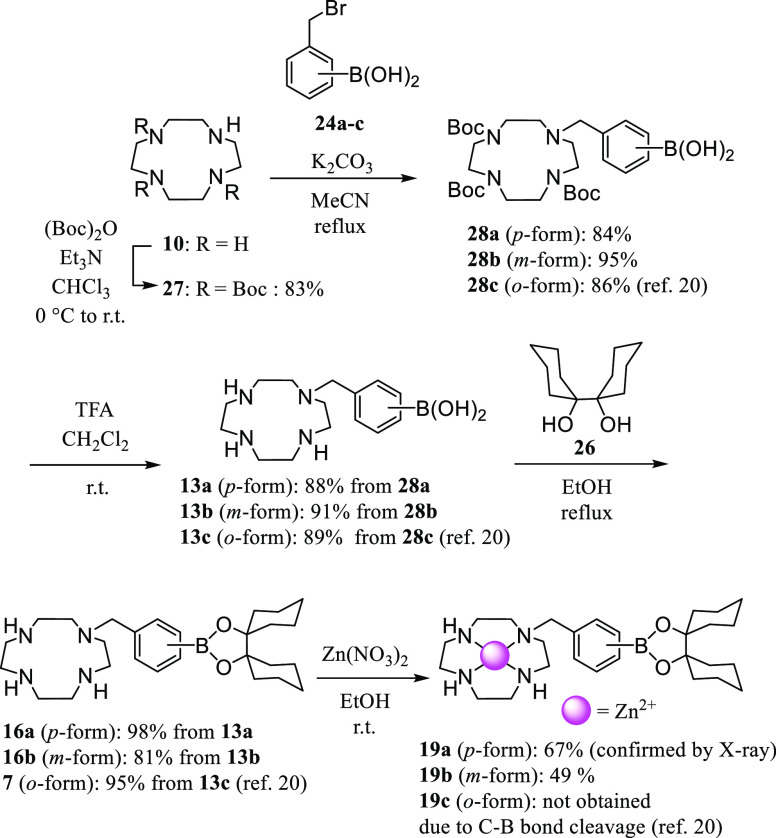
Synthesis of **13a**,**b**, **16a**,**b**, **7**, and **19a**,**b**

**Scheme 8:**
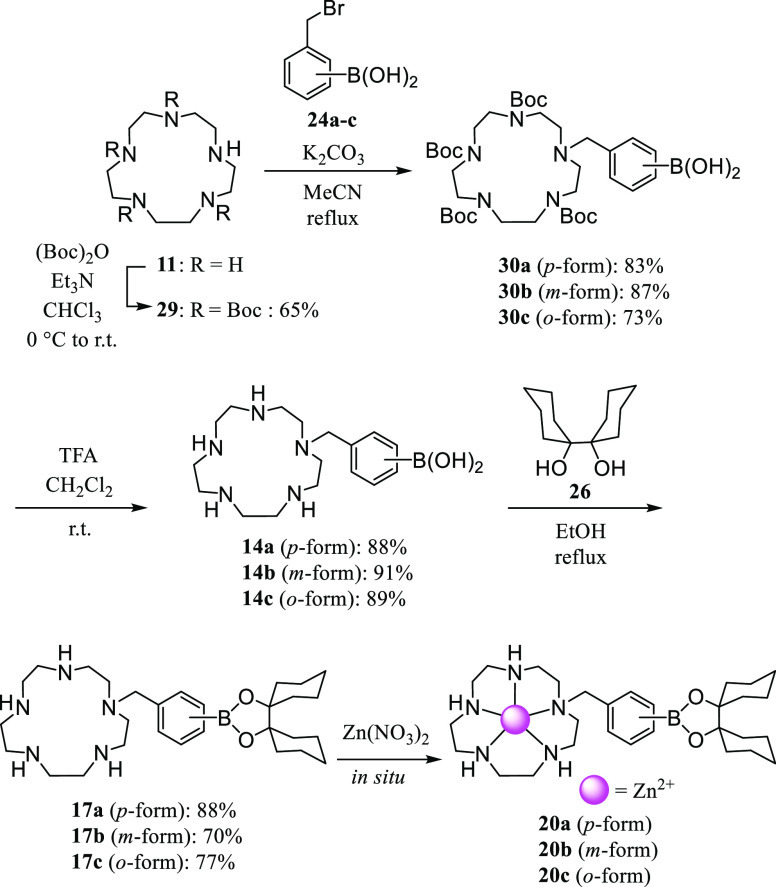
Synthesis of **14a**–**c**, **17a**–**c**, and **20a**–**c**

The synthesis of the macrocyclic polyamine derivatives is shown in Schemes 6–8. The boron-containing BNCT agents were initially synthesized using naturally abundant ratio of boron (^10^B/^11^B = 19.9/80.1), in order to evaluate their intracellular uptake, from which more potent candidates were selected and the corresponding ^10^B-enriched compounds were synthesized for use in BNCT experiments.

The 9-membered macrocyclic polyamine **9** ([9]aneN_3_)^32^ was treated with (Boc)_2_O to give **23**,^33^ which was then reacted with 4-(bromomethyl)phenylboronic acid **24a**(34) to afford **25a** (Scheme 6). After removing the Boc groups of **25a** by treatment with trifluoroacetic acid (TFA) to give **12a** as the 2TFA salt, the reaction of **12a** with bicyclohexyl-1,1′-diol **26**(35) gave **15a**. The synthesis of the *m*-isomer **15b** was carried out in a similar manner.^36^ The *o*-isomers of **12** and **15** (**12c** and **15c**) were not obtained, due to the cleavage of their C–B bonds in aqueous solution even in the absence of metal ions. The complexation of **15a** and **15b** with Zn^2+^ was conducted *in situ* before the biological evaluation.

The synthesis of the 12-membered tetraamine (cyclen) ([12]aneN_4_) derivatives **16a**,**b** and the 15-membered pentaamine ([15]aneN_5_) derivatives **17a**–**c** was carried out, as shown in Schemes 7 and 8.^32−39^ The deprotection of **28a** and **30a**–**c** with TFA afforded **13a** and **14a**–**c** 2TFA and 3TFA salts, respectively, as determined by elemental analysis.

**Figure 1:**
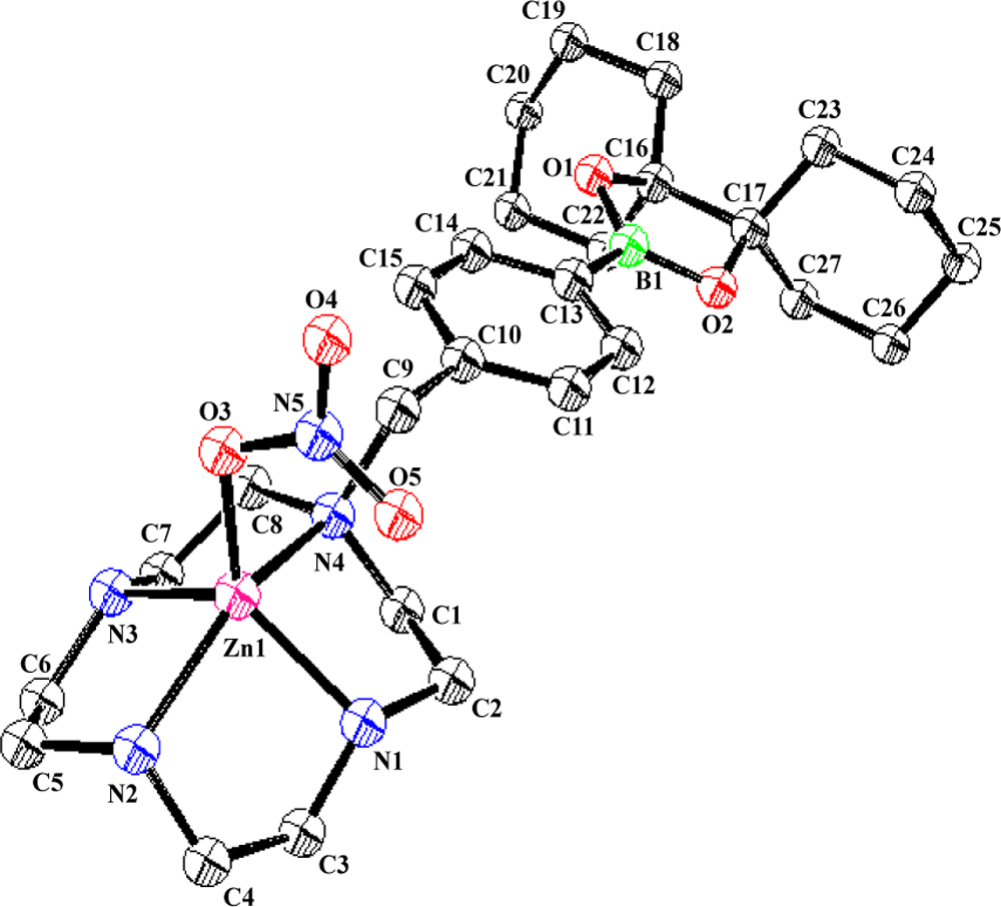
ORTEP drawing of **19a** with a Zn^2+^—bound NO_3_^–^. Selected bond lengths: Zn(1)–N(1) 2.059 Å, Zn(1)–N(2) 2.167 Å, Zn(1)–N(3) 2.091 Å, Zn(1)–N(4) 2.962 Å, Zn(1)–O(3) 1.999 Å, C(13)–B(1) 1.561 Å, B(1)–O(1) 1.363 Å, and B(1)–O(2) 1.366 Å. One external nitrate anion, ethanol, and hydrogen atoms were omitted for clarity.

The Zn^2+^ complexes of **16a** and **16b** (**19a** and **19b**) were isolated and those of **17a**–**c** (**20a**–**c**) were prepared *in situ* for use in biological experiments. The structure of **19a** was confirmed by a single-crystal X-ray structure analysis, as shown in Figure 1. The Zn^2+^ complex of the *o*-form **7** was not obtained due to the carbon–boron bond cleavage that occurred upon complexation with Zn^2+^, as previously described.^20^ In contrast, the C–B bond in **20c** (Zn^2+^–**17c** complex) was hydrolyzed very slowly (approximate half-life is 24 h) as observed by ^11^B-NMR, possibly due to the higher p*K*_a_ value of the Zn^2+^-bound water in the Zn^2+^–[15]aneN_5_ complex than that of **19c**, which is a Zn^2+^ complex of the [12]aneN_4_-type ligand **7**.^39^

### Evaluation of the Cytotoxicity of Boron-Containing Macrocyclic Polyamine Derivatives against HeLa S3, A549, and IMR-90 Cells

The cytotoxicity of the boron-containing macrocyclic polyamine derivatives **7**, **12**–**17**, and their corresponding Zn^2+^ complexes **18**–**20** against HeLa S3 (human cervical carcinoma), A549 (human caucasian lung carcinoma), and IMR-90 (normal human fibroblast) cells was examined by an MTT (3-(4,5-dimethylthiazol-2yl)-2,5-diphenyltetrazolium bromide) assay in comparison with those of BSH (**1**) and BPA–d-fructose complex (**2**). The cells (1 × 10^4^ cells/well) were incubated with boron compounds **1**, **2**, **7**, and **12**–**20** (0–200 μM) in culture medium containing 10% fetal bovine serum (FBS) for 24 h at 37 °C under 5% CO_2_ and then treated with the MTT reagent to evaluate cell viability.

The results are presented in Figures S1–S3 in the Supporting Information, and the IC_50_ values of these agents are summarized in Table 1. The findings indicated that **7** and **12**–**20** are somewhat more toxic than **1** and **2**, and that **15a**, **16a**, and **17a** are more toxic than **12a**, **13a**, and **14a**, possibly due to the hydrophobicity of the boronic acid ester group. It should be noted that the cytotoxicity of Zn^2+^ complex **19b**, **20b**, and **20c** is lower than the corresponding Zn^2+^-free ligands **16b**, **17b**, and **17c**, while the Zn^2+^-free ligands **15a**, **15b**, **16a**, **17a**, and their Zn^2+^ complexes **18a**, **18b**, **19a**, and **20a** have a similar toxicity. The similar toxicity between **15a** and **18a** and **15b** and **18b** would be due to the weak Zn^2+^ complexation of **15a** and **15b**, which have only three nitrogen atoms in the [9]aneN_3_ ring group.

**Table 1:** IC_50_ Values of Boron Compounds **1**, **2**, **7**, and **12**–**20** [0–200 μM] against HeLa S3, A549, and IMR-90 Cells after the Treatment for 24 h

compound	HeLa S3	A549	IMR-90	compound (Zn^2+^ complexes)	HeLa S3	A549	IMR-90
**1**	>200	>200	>200				
**2**	>200	>200	>200				
**7**	100 ± 9	162 ± 7	108 ± 5				
**12a**	>200	>200	>200				
**13a**	>200	>200	>200				
**14a**	>200	>200	>200				
**15a**	131 ± 11	151 ± 3	83 ± 5	**18a**	112 ± 3	155 ± 2	130 ± 9
**15b**	>200	>200	187 ± 14	**18b**	>200	>200	162 ± 5
**16a**	112 ± 4	>200	94 ± 6	**19a**	148 ± 3	139 ± 11	95 ± 16
**16b**	163 ± 1	128 ± 9	135 ± 2	**19b**	>200	>200	>200
**17a**	>200	>200	151 ± 8	**20a**	>200	>200	129 ± 16
**17b**	22 ± 1	34 ± 1	18 ± 3	**20b**	71 ± 1	>200	32 ± 5
**17c**	65 ± 2	117 ± 10	35 ± 3	**20c**	138 ± 6	197 ± 21	83 ± 8

### Intracellular Uptake of Boron-Containing Macrocyclic Polyamine Derivatives into HeLa S3, A549, and IMR-90 Cells, as Determined by Inductively Coupled Plasma Mass Spectrometry

**Figure 2:**
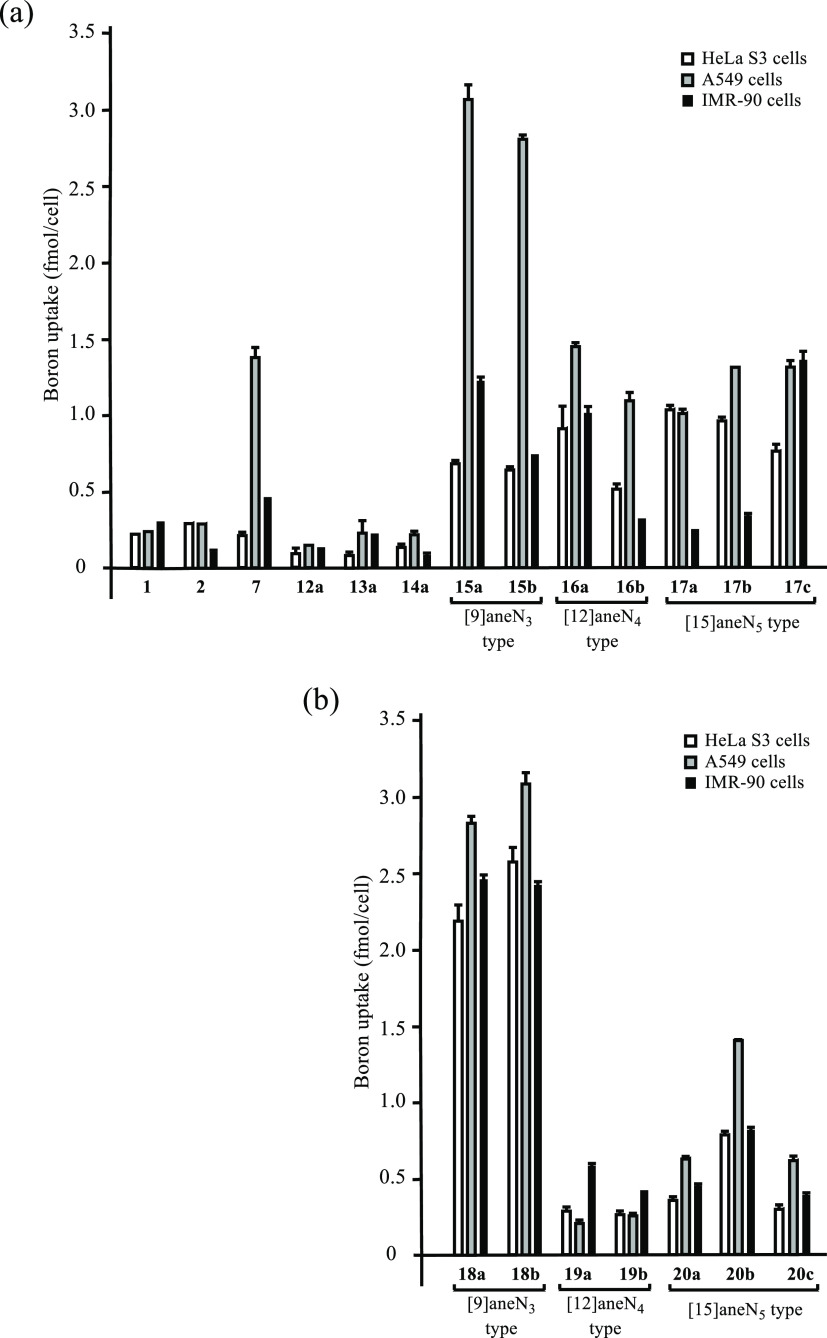
Comparison of intracellular boron atoms against HeLa S3 (open bars), A549 (shaded bars), and IMR-90 (closed bars) cells as determined by ICP–MS. All cells were treated with boron compounds **1**, **2**, **7**, **12**–**17** (a), and **18**–**20** (b) (30 μM) in culture medium at 37 °C for 24 h. Data represent the mean ± standard deviation (SD) of at least three replicates.

**Scheme 9:**
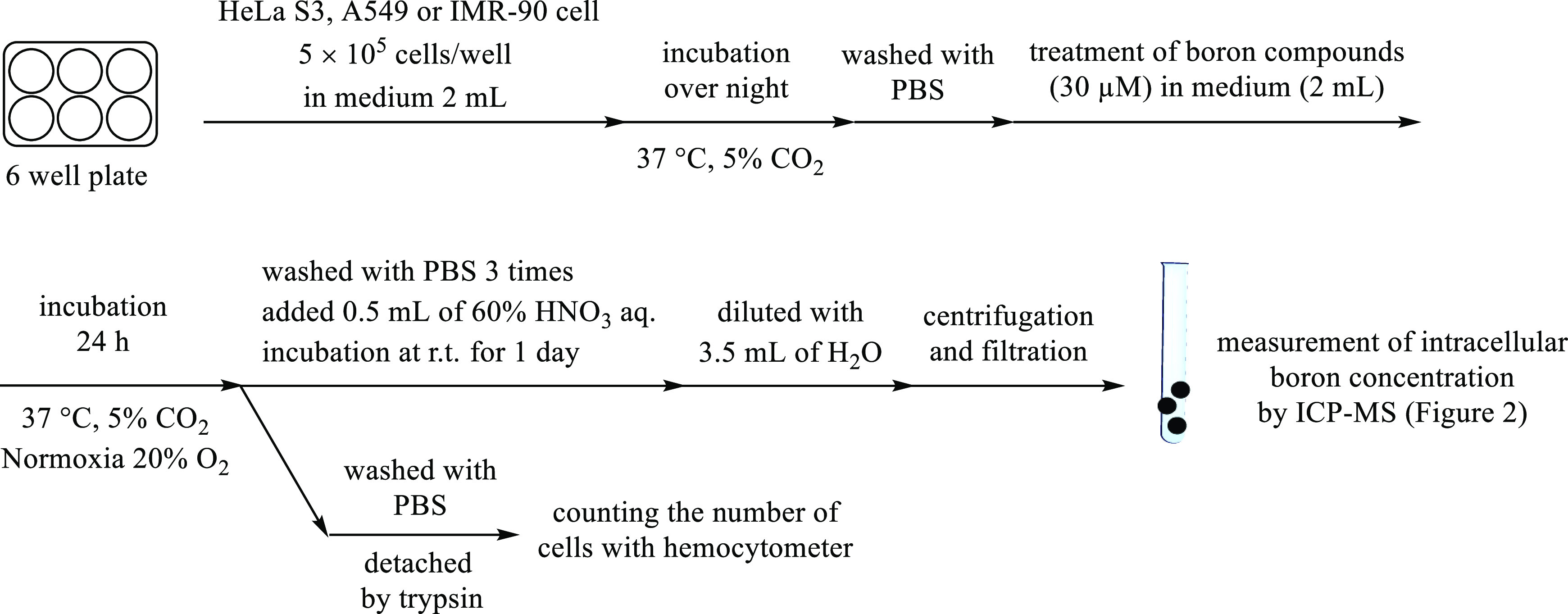
Typical Procedure Used for Measuring the Intracellular Uptake of Boron Compounds in Living Cells

The intracellular uptake of the boron compounds into HeLa S3, A549, and IMR-90 cells was evaluated by inductively coupled plasma mass spectrometry (ICP–MS), as shown in Scheme 9. The cells (5 × 10^5^ cells/well) were seeded on 6-well plates and incubated in culture medium containing 10% FBS for 1 day at 37 °C in a 5% CO_2_ environment (20% O_2_) and then treated with boron compounds **1**, **2**, **7**, and **12**–**20** (30 μM) under same conditions. This concentration (30 μM) of **1**, **2**, **7**, and **12**–**20** (lower concentrations are better to reduce their toxicity) was carefully determined based on the consideration of a balance between their IC_50_ values (toxicity) and intracellular uptake values that are listed in Table 1 and Figure 2. After incubating the cells for 24 h, they were washed with PBS and broken down with nitric acid overnight, and the amount of boron atoms (total amount of ^10^B and ^11^B) was quantitatively determined by ICP–MS and normalized as the amount of per cell because some compounds have weak toxicity.

As shown in Figure 2a, the intracellular uptake of **7** and **15**–**17** is higher than that of reference compounds BSH **1** comprised of twelve ^10^B and BPA **2**, possibly because cell-membrane permeability is improved by their boronic acid ester group. In addition, it was found that intracellular uptake of the 9-membered triamine derivatives **15a**,**b** and **18a**,**b** into A549 cells was higher than **16a**,**b** and **17a**–**c**, and their Zn^2+^ complexes **19a**,**b** and **20a**–**c** exhibited a lower intracellular uptake (Figure 2b), suggesting that the 9-membered triamine group in **15a** and **15b** is better for the intracellular uptake into A549 cells.

**Figure 3:**
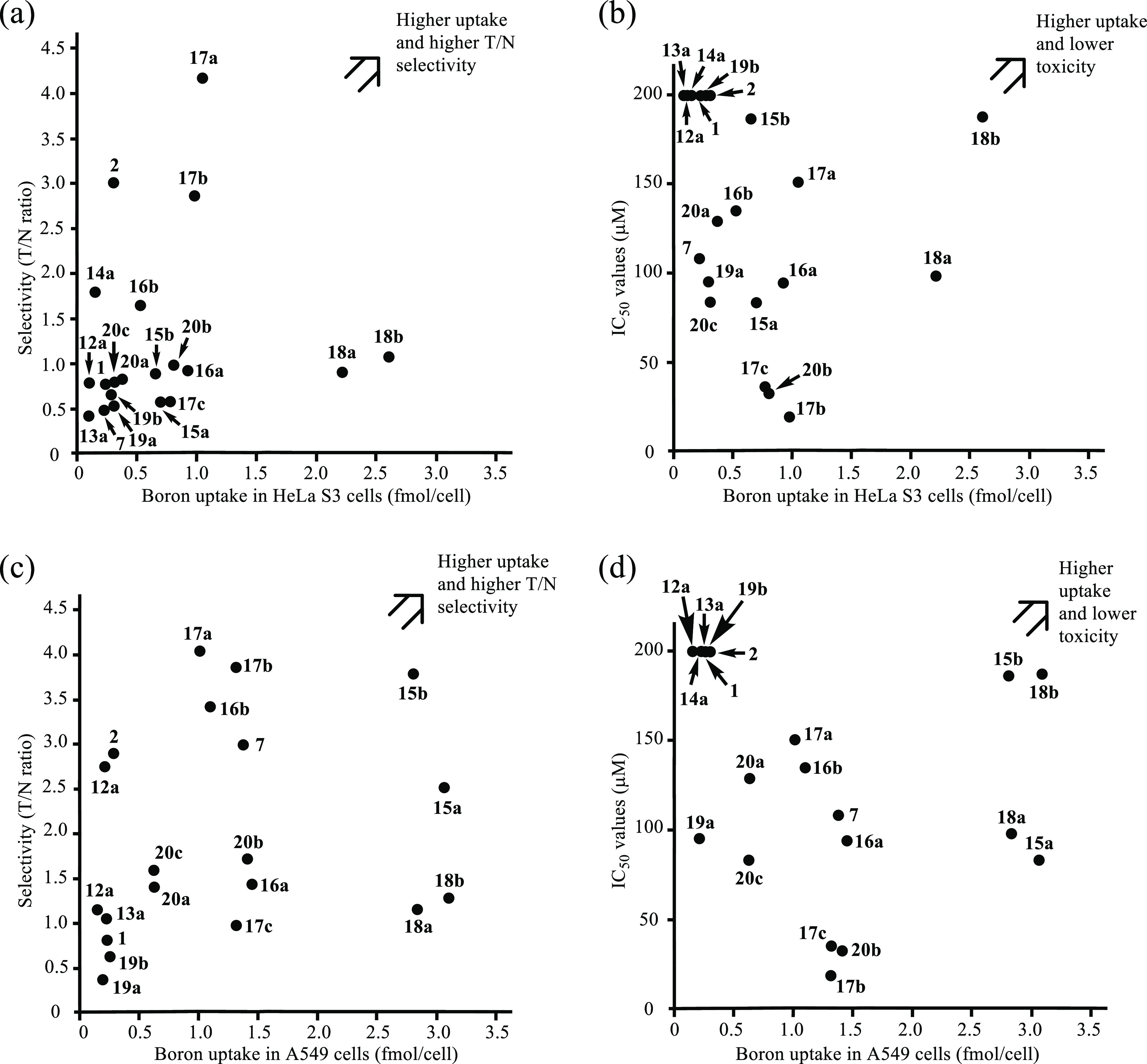
Intracellular boron uptake-T/N selectivity profiles (a,c) and intracellular boron uptake-IC_50_ value against normal cell profiles (b,d) of boron compounds **1**, **2**, **7**, and **12**–**20**. (a,c) Selectivity (T/N ratio) to HeLa S3 cells (a) and A549 cells (c) were calculated from the results for the intracellular uptake of the boron compounds into HeLa S3 and A549 cells in comparison to the uptake into IMR-90 cells, respectively. (b,d) IC_50_ values (μM) of boron compounds **1**, **2**, **7**, and **12**–**20** against IMR-90 cells and boron uptake (fmol/cell) into HeLa S3 cells (b) and A549 cells (d).

The tumor/normal cell (T/N) ratios with respect to the intracellular uptake of the boron compounds (**1**, **2**, **7**, and **12**–**20**) were calculated using eq 1, and their intracellular boron uptake (in HeLa S3 cells and A549 cells)-T/N ratio profiles are shown in Figure 3a,c. The boron uptake-IC_50_ value (indicating the toxicity) profiles are plotted in Figure 3b (HeLa S3 cells) and 3d (A549 cells). These data suggest that **17a** has a higher boron uptake (>2.5 fmol/cell) and T/N selectivity (ca. 4) and a rather low toxicity against HeLa S3 cells (Figure 3a,b) and that **15b**, **16b**, and **17a** exhibit better boron uptake, higher T/N ratios (over 3), and lower toxicity against A549 cells and normal cells (IC_50_ > 100 μM) (Figure 3c,d), although the reasons for their selective uptake to cancer cells are yet to be studied.
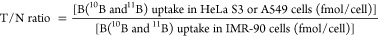
1

**Scheme 10:**
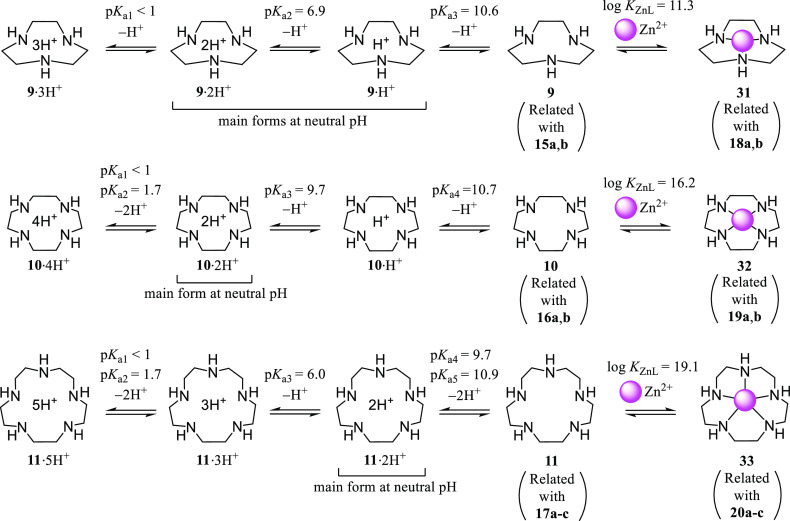
Reported Deprotonation Constants (p*K*_a_) of Macrocyclic Polyamines **9**–**11**^24−26^ and Stability Constants (log *K*_ZnL_) of Their Zn^2+^ Complexes **31**–**33** in Aqueous Solution at 25 °C^27^

Concerning the relationship of these data and the protonation properties of the aforementioned boron-macrocyclic polyamine conjugates, the deprotonation constants (p*K*_a_ values) of unmodified macrocyclic polyamines **9**, **10**, and **11** are summarized in Scheme 10.^24−26^ It is likely that the major forms of **9** (the amine moieties of **15a**,**b**) at neutral pH are diprotonated (**9**·2H^+^) and monoprotonated (**9**·H^+^) forms, and those of **10** (the amine moieties of **16a**,**b**) and **11** (the amine moieties of **17a**–**c**) are diprotonated forms (**10**·2H^+^ and **11**·2H^+^, respectively). These findings regarding the intracellular uptake of **15a**,**b**, **16a**,**b**, and **17a**–**c** (Figure 2) suggest that the diprotonated and/or monoprotonated forms of these boron–polyamine conjugates are preferable for effective intracellular uptake and that the monoprotonated form might be more favorable. The intrinsic stability constants (log *K*_ZnL_) of the Zn^2+^ complexes **31**–**33** are also described in Scheme 10. The similar intracellular uptake of [9]aneN_3_-type **15a**,**b** and **18a**,**b** in A549 cells and higher uptake of **18a**,**b** than that of **19a**,**b** and **20a**–**c** (Figure 2) can be explained by a smaller log *K*_ZnL_ value for **31** (Zn^2+^–**9** complex) than those for **32** (Zn^2+^–**10** complex) and **33** (Zn^2+^–**11** complex) (less stability of **31** than **32** and **33**), although the reasons for higher intracellular uptake of **18a**,**b** than **15a**,**b** in HeLa S3 cells and IMR-90 cells are yet to be studied. The relationship of these complexation properties and the results of BNCT experiments of **15**, **16**, and **17** will be discussed below.

Consideration of protonation/deprotonation situations in Scheme 10 suggest that [9]aneN_3_ (**9**) and [15]aneN_5_ (**11**) would exist as **9**·2H^+^ and **11**·3H^+^ forms as well as **9**·H^+^ and **11**·2H^+^ forms, respectively, under (slightly) acidic conditions in cancer cells, so that exclusion of these drugs form the cells through the hydrophobic cell membrane would be somewhat disturbed. This point might be one of advantages of these boron–polyamine agents. It is unlikely that **9**, **10**, and **11** exist as **9**·3H^+^, **10**·3H^+^, **10**·4H^+^, **11**·4H^+^, and **11**·5H^+^ forms, respectively, under physiological conditions, because their p*K*_a1_ values (and p*K*_a2_ values for **10** and **11**) are very low (less than 2).

### Effect of Temperature and Inhibitors on the Intracellular Uptake of Boron Compounds

**Figure 4:**
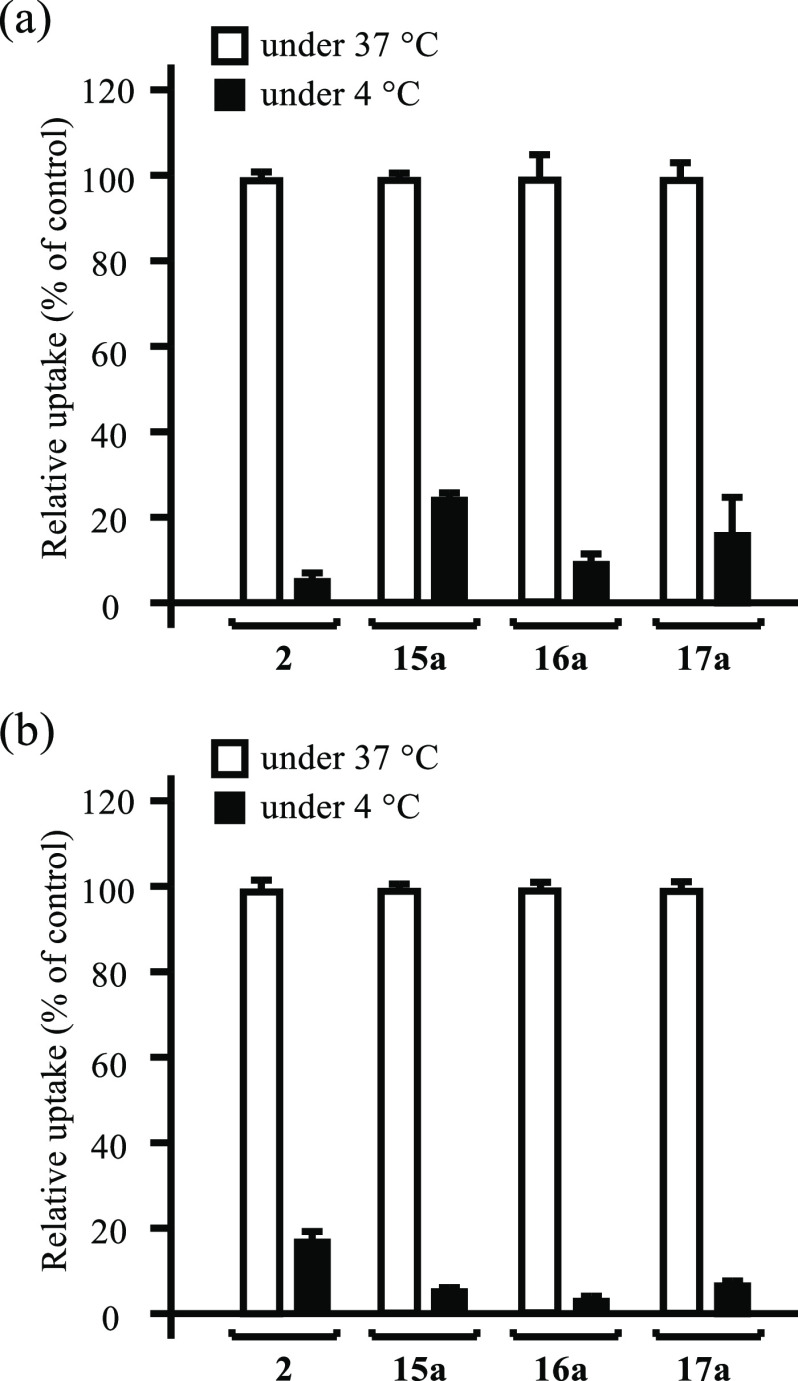
Effect of low temperature on the intracellular uptake of boron compounds **2** and **15a**–**17a** (30 μM) into HeLa S3 (a) and A549 cells (b) at 37 °C (open bars) or 4 °C (closed bars) for 1 h. Data represent the mean ± SD of at least three replicates.

The mechanism responsible for the intracellular uptake of **15a**, **16a**, and **17a** into HeLa S3 and A549 cells was examined. As shown in Figure 4a,b, the intracellular uptake of **15a**, **16a**, and **17a** was inhibited to a considerable extent at 4 °C, suggesting that the transfer of **15a**, **16a**, and **17a** into the cells is due to an energy-dependent process.

**Figure 5:**
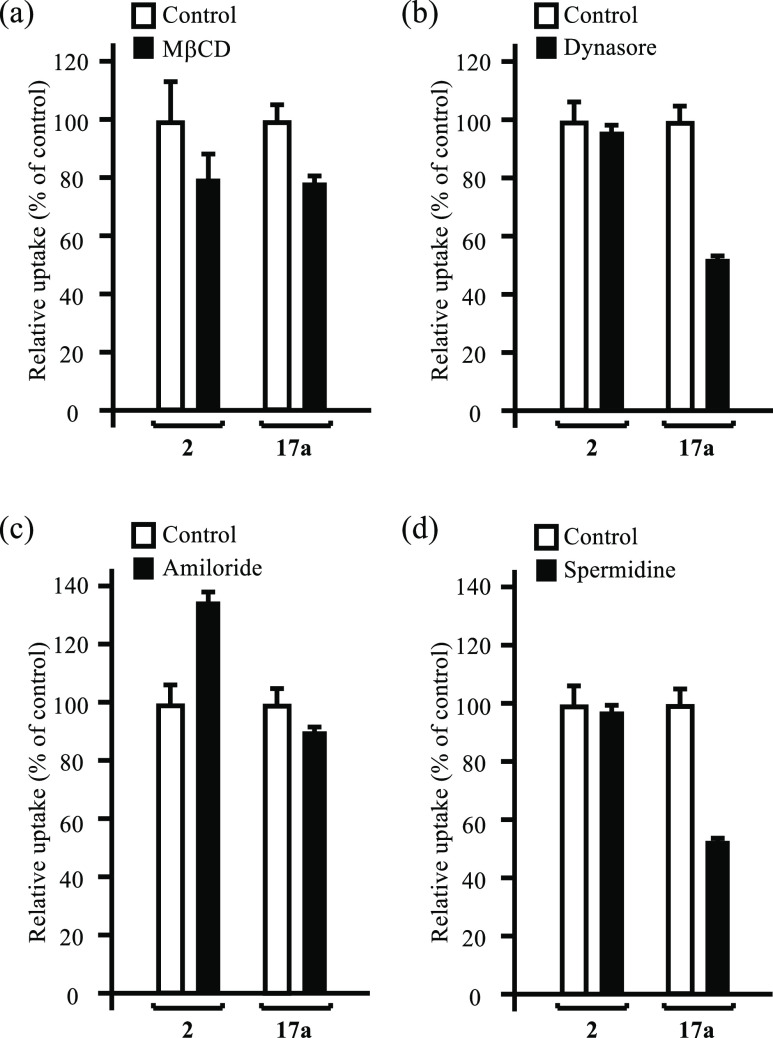
Relative uptake of **2** and **17a** (30 μM) into HeLa S3 cells in the absence (open bars) and presence of inhibitors (closed bars), 1.5 mM of MβCD (a), 80 μM of dynasore (b), 2 mM of amiloride (c), and 2 mM of spermidine **3** (d). After pretreatment with the inhibitors for 1 h, the cells were incubated with **2** and **17a** at 37 °C for 1 h in the presence of inhibitors. Data represent the mean ± SD of at least three replicates.

It is known that the PTS in mammalian cells is associated with the endocytosis pathway of linear polyamines such as spermidine **3** and spermine **4** (Scheme 2).^18^ In addition, methyl-beta-cyclodextrin (MβCD) was reported to inhibit the caveola-endocytosis pathway due to the depletion of cholesterol,^40^ and dynasore and amiloride are used as inhibitors of clathrin-endocytosis^41^ and micropinocytosis,^42^ respectively (the chemical structures of these inhibitors are shown in Scheme S1 of the Supporting Information). As shown in Figure 5, the intracellular uptake of **17a** into HeLa S3 cells is inhibited to a considerable extent by dynasore and spermidine **3**, suggesting that **17a** is transferred into the cells via the clathrin-endocytosis pathway, possibly including PTS.^18^ A similar inhibitory effect of spermidine on the intracellular uptake of **17a** into A549 cells was observed, as shown in Figure S4 in the Supporting Information. We assume that the weak inhibition of the uptake of **2** and **17a** by MβCD is due to the inclusion of these boron compounds in the inner cavity of MβCD. It is reported that amiloride inhibits the Na^+^/H^+^ exchanger and hence lower the intracellular Na^+^ concentration. It is assumed that this Na^+^ deficiency would be compensated by the Na^+^ uptake via sodium-dependent amino acid transporters such as ATB^0,+^ (amino acid transporter system B^0,+^) that had been reported to mediate the co-transport of Na^+^ with phenylalanine analogue **2**.^4c^ This assumption may explain the increased intracellular uptake of **2** in the presence of amiloride.

### Evaluation of the Anti-tumor Effect of the Selected Boron-Containing Macrocyclic Polyamine Derivatives with Thermal Neutron Irradiation by a Colony Formation Assay

**Scheme 11:**
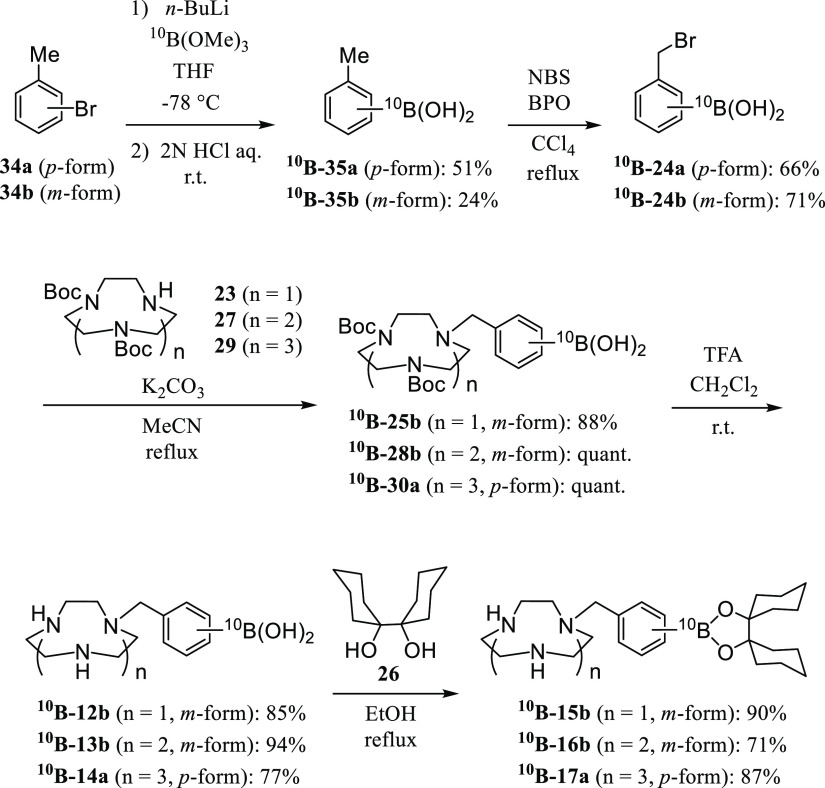
Synthesis of ^10^B-Enriched **15b**, **16b**, and **17a** (^**10**^**B-15b**, ^**10**^**B-16b**, and ^**10**^**B-17a**)

Based on the aforementioned results, we decided to choose **15b**, **16b**, and **17a** for the BNCT, in which ^10^B and ^11^B are contained in a natural abundance ratio (^10^B/^11^B = 19.9/80.1) and synthesized the corresponding ^10^B-enriched compounds ^**10**^**B-15b**, ^**10**^**B-16b** and ^**10**^**B-17a**, as shown in Scheme 11.

The ^10^B-enriched forms of **24a** and **24b** (^**10**^**B-24a** and ^**10**^**B-24b**) were prepared by the reaction of **34a**,**b** with ^10^B-enriched trimethyl borate (>99.5% of ^10^B), followed by hydrolysis with aqueous HCl to give ^**10**^**B-35a** and ^**10**^**B-35b** and bromination with *N*-bromosuccinimide (NBS). The reaction of ^**10**^**B-24a** and ^**10**^**B-24b** with **23**, **27**, and **29** and the following conversions were conducted as described in Schemes 6–8 to obtain ^**10**^**B-15b**, ^**10**^**B-16b**, and ^**10**^**B-17a**, respectively.

**Figure 6:**
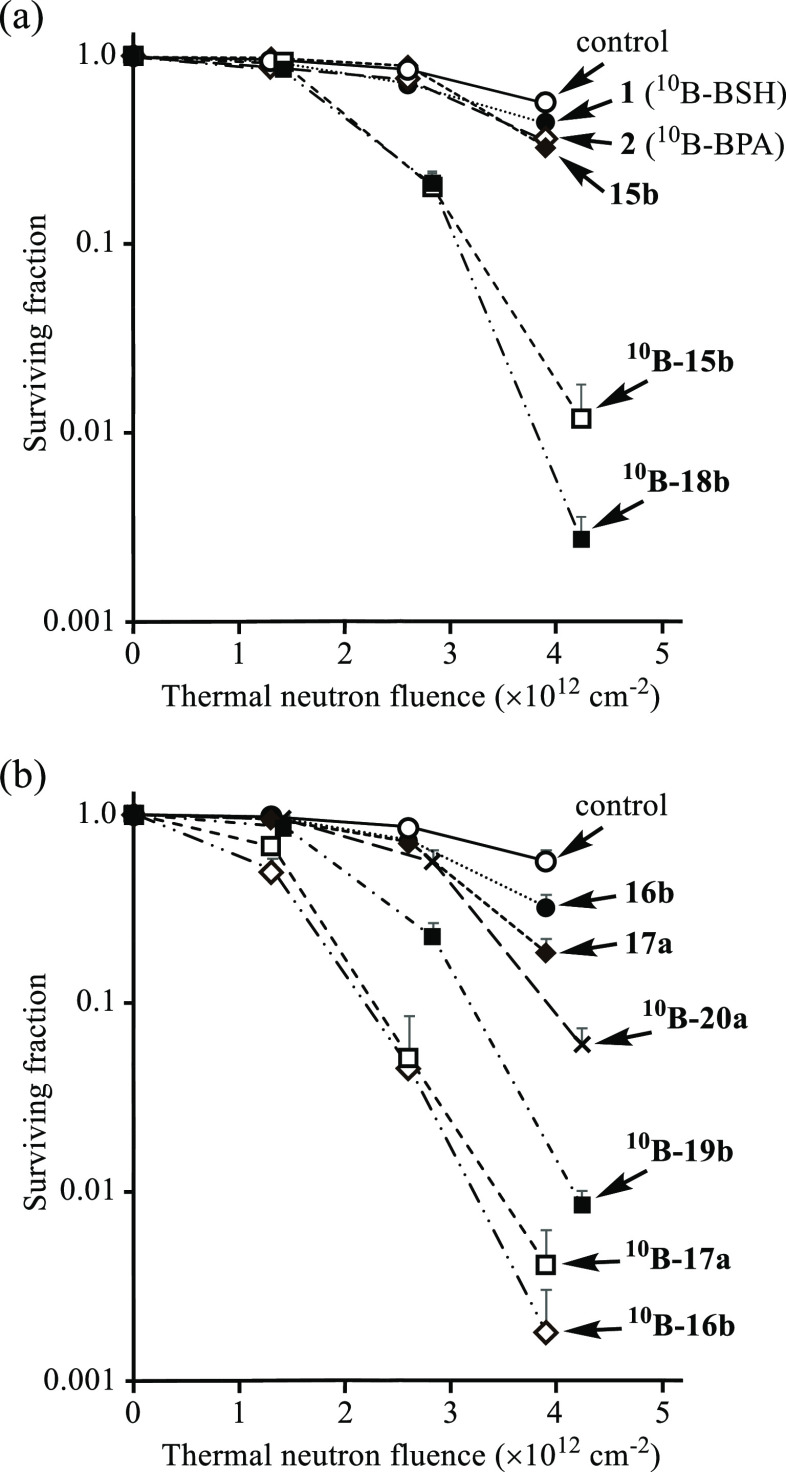
Anti-tumor effect of boron compounds **1**, **2**, **15b**, ^**10**^**B-15b**, **16b**, ^**10**^**B-16b**, **17a**, ^**10**^**B-17a**, ^**10**^**B-18b,**^**10**^**B-19b**, and ^**10**^**B-20a** (30 μM) against A549 cells was examined by a colony formation assay: (a) control (in the absence of boron compound) (○), **1** (●), **2** (◇), **15b** (◆), ^**10**^**B-15b** (□), and ^**10**^**B-18b** (■). (b) Control (○), **16b** (●), ^**10**^**B-16b** (◇), **17a** (◆), and ^**10**^**B-17a** (□), and ^**10**^**B-19b** (■), and ^**10**^**B-20a** (×). After treatment with the boron compound for 24 h, the cells were irradiated with thermal neutrons for 0, 15, 30, and 45 min and then incubated without neutron irradiation for 7 days. Averaged thermal neutron flux was 1.4 × 10^9^ n/cm^2^·s for control (in the absence of boron compound), **1**, **2**, **15b**, **16b**, ^**10**^**B-16b**, **17a**, and ^**10**^**B-17a** and 1.6 × 10^9^ n/cm^2^·s for ^**10**^**B-15b**, ^**10**^**B-18b**, ^**10**^**B-19b**, and ^**10**^**B-20a**, respectively. The survival fraction was determined by ImageJ-plugin Colony Area. Data represent the mean ± SD of at least three replicates.

**Scheme 12:**
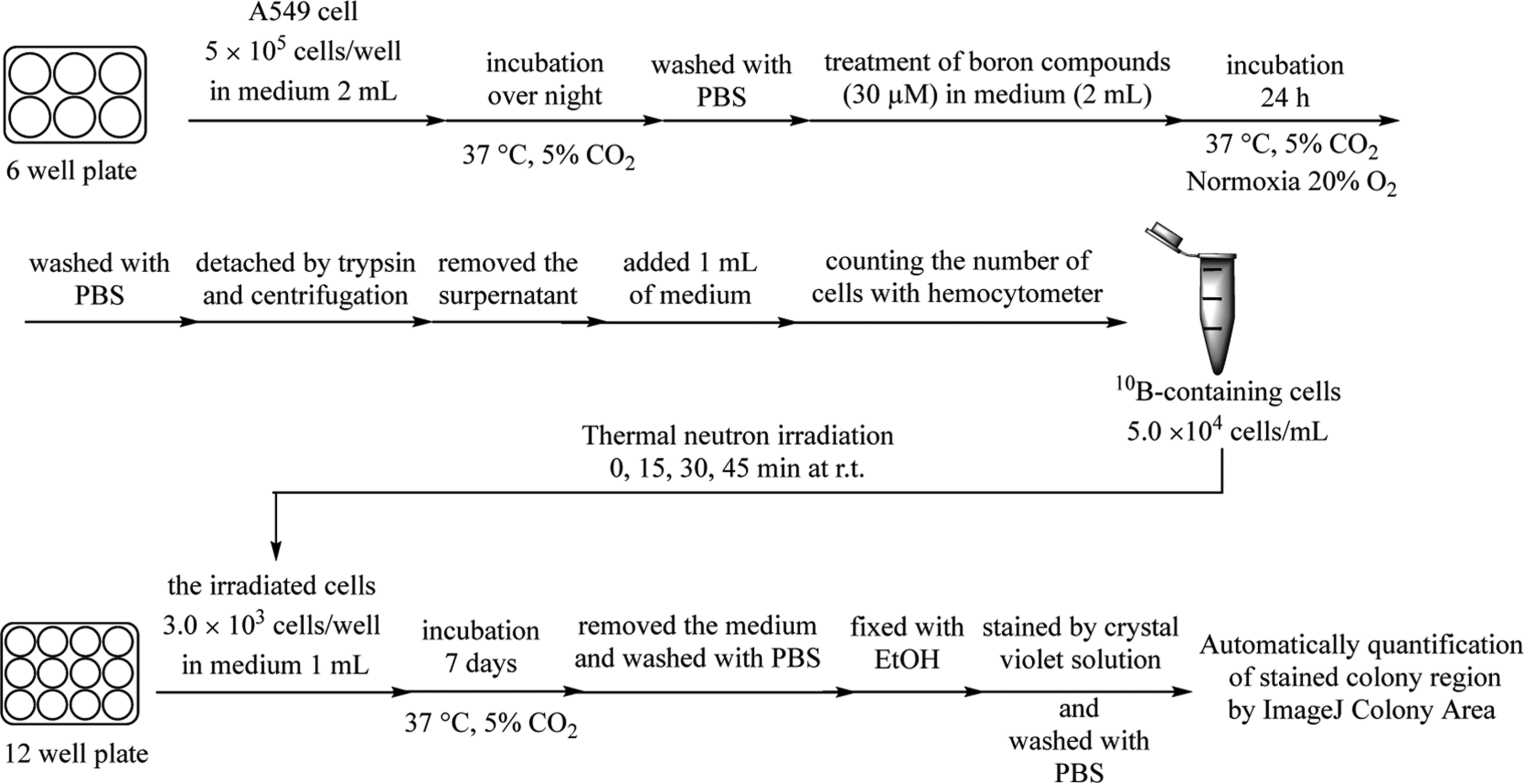
Evaluation of the Anti-tumor Effect of Boron Compounds in an In Vitro BNCT Study

BNCT experiments using A549 cells in the presence of the aforementioned B-containing drugs (^10^B/^11^B and ^10^B-enriched compounds) were conducted at the Institute for Integrated Radiation and Nuclear Science, Kyoto University (KURNS). As shown in Scheme 12, A549 cells were incubated with the boron compounds (30 μM) for 24 h and suspensions (5 × 10^4^ cells/mL) of these cells were irradiated with thermal neutrons [average thermal neutron flux: (1.5 ± 0.1) × 10^9^ n/cm^2^·s] at room temperature for various times (0, 15, 30, and 45 min). The irradiated cells were seeded on the 12 well plate (3 × 10^3^ cells/well), incubated for 7 days, fixed with EtOH, and stained with crystal violet to produce visualizable images (Figure S5 in the Supporting Information). The surviving fractions were calculated as the stained colony area using the “ImageJ-plugin Colony Area”^43^ software and normalized by comparing the results with those for non-irradiated cell samples (Figure 6).

**Figure 7:**
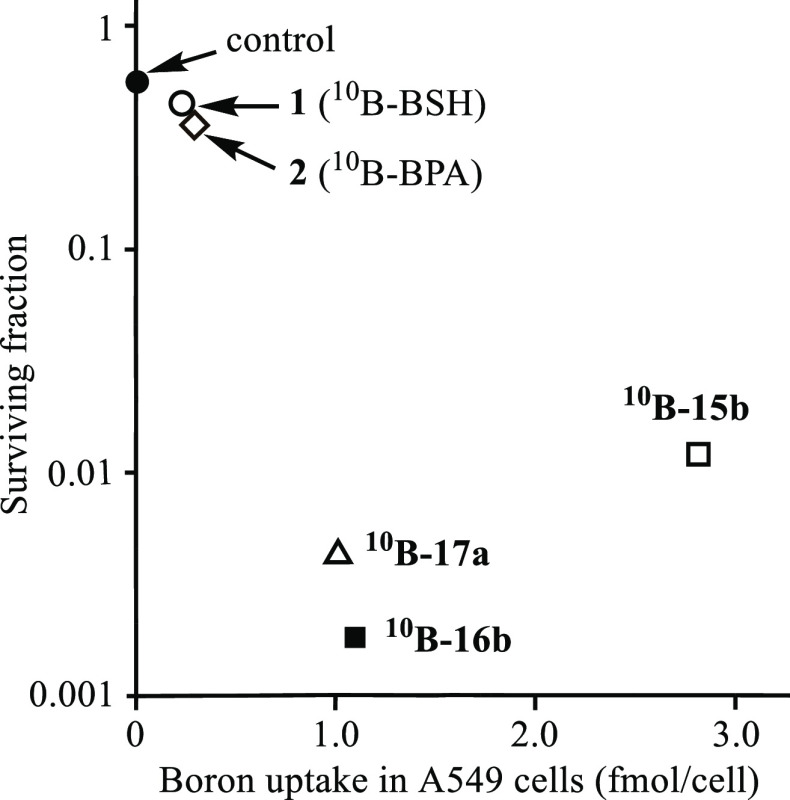
Relationship between the intracellular uptake of boron compounds **1** (○), **2** (◇), ^**10**^**B-15b** (□), ^**10**^**B-16b** (■), ^**10**^**B-17a** (△) (30 μM) and control (in the absence of boron compound) (●) into A549 cells after incubation for 24 h and their BNCT effect (survival fractions after irradiation with thermal neutrons for 45 min; thermal neutron fluence: 4.1 ± 0.1 × 10^12^ n/cm^2^).

The results for the anti-tumor effect of boron compounds against A549 cells are summarized in Figure 6, which suggests the following points:

(1) The cytotoxic activity of ^**10**^**B-15b**, ^**10**^**B-16b**, and ^**10**^**B-17a** against A549 cells is higher than that for **1** (^10^B-BSH) and **2** (^10^B-BPA).
(2) The cytotoxic activity of the ^10^B-enriched analogues is more potent than that of the ^10^B/^11^B derivatives (^**10**^**B-15b** vs **15b**, ^**10**^**B-16b** vs **16b**, and ^**10**^**B-17a** vs **17a**) apparently due to the enrichment of ^10^B.
(3) The BNCT activity of ^**10**^**B-18b**, ^**10**^**B-19b**, and ^**10**^**B-20a**, which are Zn^2+^ complexes of ^**10**^**B-15b**, ^**10**^**B-16b**, and ^**10**^**B-17a**, is also displayed in Figure 6. It was found that metal-free ^**10**^**B-16b** and ^**10**^**B-17a** exhibit a higher BNCT effect than ^**10**^**B-19b** and ^**10**^**B-20a**, possibly because of their higher intracellular uptake than that of stable ^**10**^**B-19b** and ^**10**^**B-20a**, which are very stable (see Figure 2 and Scheme 10).
(4) The BNCT effect of ^**10**^**B-15b** and its Zn^2+^ complex ^**10**^**B-18b** were nearly the same, possibly due to rather low stability of ^**10**^**B-18b**, as indicated by a relatively small log *K*_ZnL_ value (11.3) for **31** (Zn^2+^–**9**) in Scheme 10.
(5) The relationship between the intracellular boron uptake (from Figure 2) and the BNCT effect (from Figure 6) is summarized in Figure 7. The BNCT effect of ^**10**^**B-16b** and ^**10**^**B-17a** was more potent than that of ^**10**^**B-15b**, while the intracellular uptake of the metal-free ^**10**^**B-16b** and ^**10**^**B-17a** was lower than that of ^**10**^**B-15b**. It was confirmed that the intracellular uptake of ^10^B/^11^B forms and ^10^B-enriched forms of **15b**, **16b**, and **17a** was almost the same, respectively (data are not shown).
(6) As presented in Figures 4 and 5, intracellular uptake of boron-containing macrocyclic polyamines is considerably inhibited at 4 °C and in the presence of endocytosis inhibitor and spermidine. Besides, the BNCT effect of ^10^B-enriched agents is not parallel to their intracellular uptake, as shown in Figure 7, which suggests their close interaction with DNA in living cells. Therefore, it is likely that B-macrocycles are transferred into living cells via an energy-dependent process such as endocytosis and then make a close contact to DNA, resulting in an efficient BNCT effect, although the possibility of the partial distribution of these boron agents in the cell membrane cannot be denied.

**Scheme 13:**
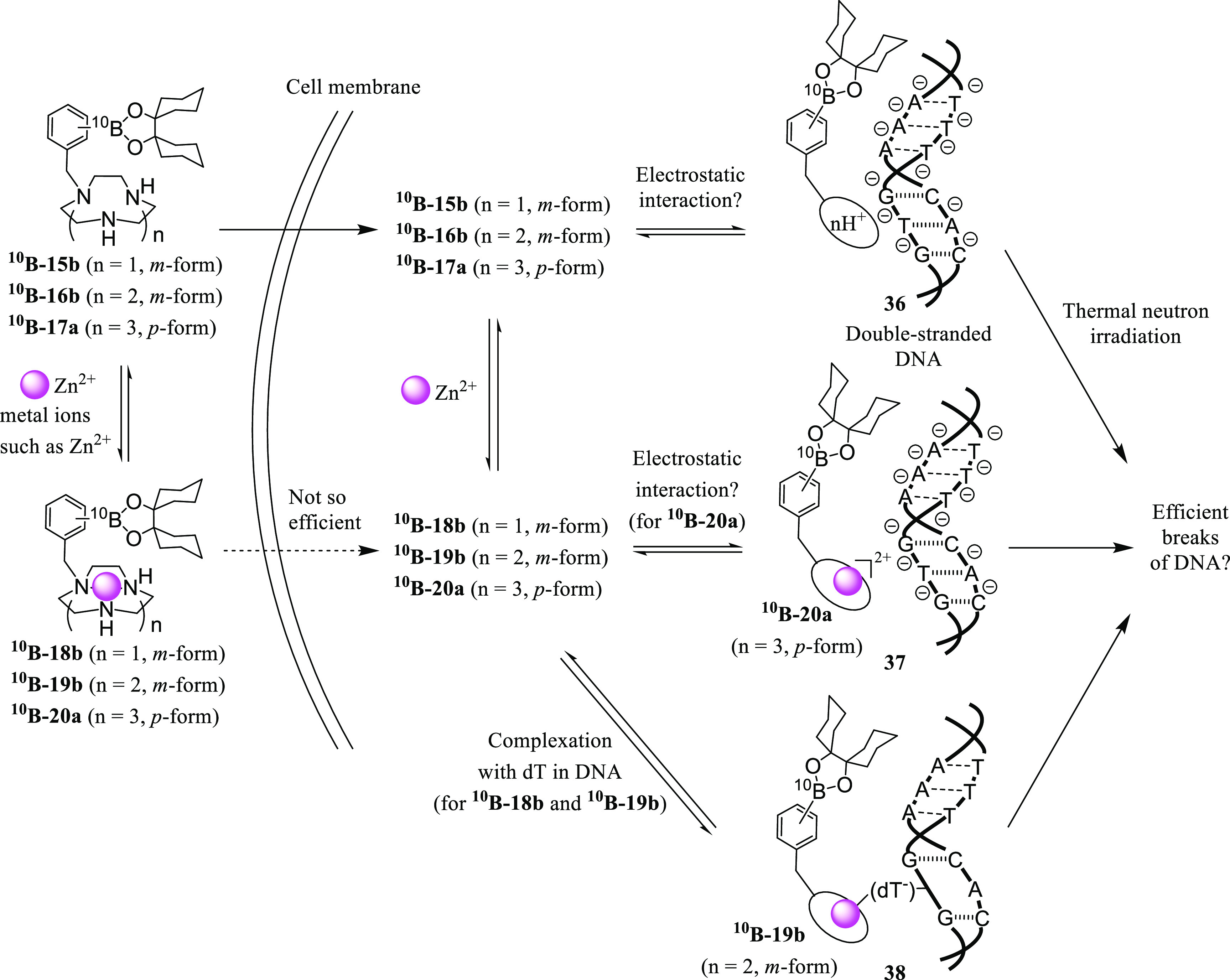
Proposed Scheme for the BNCT Effect of ^**10**^**B-15b**, ^**10**^**B-16b**, ^**10**^**B-17a**, and Their Zn^2+^ Complexes

These experimental data allow us to propose two possibilities for the BNCT effect of ^**10**^**B-15b**, ^**10**^**B-16b**, and ^**10**^**B-17a**, as presented in Scheme 13. One possible explanation would be that cytotoxicity is dependent on the close interaction of metal-free macrocyclic polyamines with DNA via the ionic interaction (**36** in Scheme 13) and the amount of double-strand breaks in DNA by ^4^He and/or ^7^Li generated by the [^10^B(n, α)^7^Li] reaction.^44^ More plausible possibility would be the breakdown of DNA via the interaction with metal complexes ^**10**^**B-18b**, ^**10**^**B-19b**, and ^**10**^**B-20a** (**37** and **38** in Scheme 13), because it is very likely that these B-containing macrocyclic polyamines would form complexes with metal cations contained in the media and/or in living cells.

As described in the Introduction (Scheme 5), we expected that the Zn^2+^ complexes ^**10**^**B-18b**, ^**10**^**B-19b**, and ^**10**^**B-20a** would interact with deprotonated dT (dT^–^) in DNA and that the DNA would be efficiently damaged upon thermal neutron irradiation (**38** in Scheme 13) (it had been reported that Cu^2+^, Ni^2+^, and Fe^2+^ complexes of cyclen negligibly interact with DNA).^31c^ These data allow us to consider that the metal-free ^**10**^**B-15b**, ^**10**^**B-16b**, and ^**10**^**B-17a** (possibly the diprotonated form, as speculated in Scheme 10) are transferred into cancer cells efficiently and form complexes with intracellular Zn^2+^ and recognize dT in DNA, resulting in an efficient BNCT effect.

In order to obtain experimental data for this hypothesis, we measured the melting temperature (*T*_m_) of the double-stranded calf-thymus DNA (ctDNA) (50 μM in phosphate) in the presence of **16b**, **19b**, **17a**, **20a**, and **3** (for the reference).^31d,31e,45^ As shown in Table 2 and Figure S6 in the Supporting Information, the *T*_m_ value of ctDNA was raised by **16b**, **17a**, and **3** (Δ*T*_m_ = +6 and +8 °C for **16b** and **17a**, respectively, at *r* = 5.0 and Δ*T*_m_ = +12 °C for **3** at *r* = 0.2, where *r* = [**16b**, **17a**, or **3**]/[ctDNA(P)]) due to stabilization of the double-stranded structure of ctDNA (**36** in Scheme 13). On the other hand, the *T*_m_ value was lowered in the presence of **19b** (Δ*T*_m_ = −6 °C at *r* = 1.0), possibly due to the destabilization of ctDNA by the interaction of its Zn^2+^–[12]aneN_4_ complex part with dT units in DNA (Scheme 5 and **38** in Scheme 13).

**Table 2:** *T*_m_ Values of ctDNA in the Presence of **3**, **16b**, **19b**, **17a**, and **20a** (*r* = [**3**, **16b**, **19b**, **17a**, or **20a**]/[ctDNA(P)]) ([ctDNA(P)] = 50 μM in Phosphate)

additive	*r*	*T*_m_ (°C)	Δ*T*_m_ (°C)
none		66	
**3**	0.1	73	+7
	0.2	78	+12
**16b**	0.1	66	
	0.5	66	
	1.0	67	+1
	5.0	72	+6
**19b**	0.1	64	–2
	0.5	62	–4
	1.0	60	–6
**17a**	1.0	69	+3
	5.0	74	+8
**20a**	0.5	71	+5
	1.0	72	+6

An interesting finding was that the *T*_m_ value was raised by **20a** (Δ*T*_m_ = +6 °C at *r* = 1.0), suggesting that **19b** and **20a** interact with ctDNA in different modes. It should be noted that coordination sites of Zn^2+^, whose general coordination number is 4–6 (or 7),^29b,39,46^ would be almost occupied by the coordination of five nitrogens from its [15]aneN_5_ ring unit of **20a** and hence Lewis acidity of the Zn^2+^ ion would be considerably reduced. Therefore, it is considered that these factors would hamper the coordination of **20a** with dT^–^ sites in DNA and that **20a** interacts with ctDNA mainly by the electrostatic interaction to stabilize the DNA double-strand, as shown in **37** of Scheme 13.^47,48^ These data support the efficient DNA damage induced by ^**10**^**B-16b**, ^**10**^**B-19b**, ^**10**^**B-17a**, and ^**10**^**B-20a** at the close position upon neutron irradiation, as proposed in Scheme 13.

## Conclusions

In conclusion, we report on the design and synthesis of boron-containing macrocyclic polyamine derivatives as novel boron delivery agents for BNCT. The results of biological studies suggest that the intracellular uptake of **15**–**17**, especially, the 9-membered triamine derivatives **15a** and **15b**, into cancer cells is higher than that of **1** and **2**, and that **15b**, **16b**, and **17a** are selectively transferred into A549 cells. The results of BNCT experiments using A549 cells in the presence of **15b**, **16b**, and **17a** including boron in a natural abundance ^10^B/^11^B ratio and their ^10^B-enriched derivatives ^**10**^**B-15b**, ^**10**^**B-16b**, and ^**10**^**B-17a** suggest that metal-free forms of these boron carriers inhibit the proliferation of A549 cells to a considerable extent after irradiation with thermal neutrons and that this inhibition is stronger than that for **1** and **2**. In addition, it was suggested that [12]aneN_4_-type ^**10**^**B-16b** and [15]aneN_5_-type ^**10**^**B-17a** exhibit a higher BNCT effect than [9]aneN_3_-type derivatives, possibly due to the formation of the corresponding Zn^2+^ complexes (^**10**^**B-19b** and ^**10**^**B-20a**) in cancer cells that would interact with DNA, although ^**10**^**B-19b** and ^**10**^**B-20a** interact with DNA in different modes. It should also be noted that macrocycles containing boron in a natural abundance ^10^B/^11^B ratio are capable of inducing cell death in BNCT to some extent and hence can be used as ^11^B MRI probes to detect their distribution in living bodies as well as BNCT agents.

We believe that these findings provide useful information for the further development of BNCT for cancer treatment. The design and synthesis of more efficient and safer (high T/N ratio) boron carriers are currently underway in our laboratory.

## Experimental Section

### General Information

All reagents and solvents were purchased at the highest commercial quality and were used without further purification. MTT was purchased from Dojindo Laboratories. Spermidine was purchased from WAKO Pure Chemical Industries Ltd. MβCD, amiloride, and ctDNA were purchased from Sigma-Aldrich. Dynasore was purchased from Tokyo Chemical Industry. ^10^B-B(OMe)_3_ was purchased from Katchem Ltd. Anhydrous tetrahydrofuran (THF) was prepared by distillation from sodium and benzophenone. All aqueous solutions were prepared using deionized water. ^1^H (300 and 400 MHz), ^13^C (100 MHz), and ^11^B (128 MHz) NMR spectra were recorded on a JEOL Always 300 (JEOL, Tokyo, Japan) and a JEOL LAMDA 400 (JEOL, Tokyo, Japan) spectrometer. Tetramethylsilane (TMS) was used as an internal reference (0 ppm) for ^1^H and ^13^C NMR measurements in CDCl_3_ and acetone-*d*_6_ and DMSO-*d*_6_. 3-(Trimethylsilyl)propionic-2,2,3,3-*d*_4_ acid sodium (TSP) was used as an internal reference (0 ppm) for ^1^H NMR measurements in D_2_O. 1,4-Dioxane was used as an internal reference (67.19 ppm) for ^13^C NMR measurements in D_2_O. ^11^B NMR spectra were measured in quartz NMR tubes using boron trifluoride diethyl ether complex (BF_3_·OEt_2_) in CDCl_3_ as an internal reference (0 ppm). IR spectra were recorded on Perkin-Elmer FTIR Spectrum 100 (ATR) (PerkinElmer, Massachusetts, USA). Melting points were measured on a Yanaco micro melting point apparatus and are uncorrected. MS measurements were performed on a Sciex X500R QTOF (AB SCIEX, Framingham, Massachusetts, USA) and Varian 910-MS (Varian Medical Systems, California, USA) spectrometer. Elemental analyses were performed on a 2400 series II CHNS elemental analyzer (PerkinElmer, Massachusetts, USA) to determine the purity (>95%) of all compounds. Isotopic purity of ^10^B were determined on ICP–MS (NexION300S, PerkinElmer, Waltham, Massachusetts, USA). Thin-layer chromatography (TLC) and silica gel column chromatography were performed using Merck Silica gel 60 F_254_ plate (Merck KGaA, Darmstadt, Germany) and Fuji Silysia Chemical FL-100D (Fuji Silysia Chemical, Aichi, Japan), Fuji Silysia Chromatorex NH-DM1020 silica gel for chromatography (Fuji Silysia Chemical, Aichi, Japan), respectively.

#### 1-[(4-Boronopheny)methyl]-4,7-bis(*tert*-butoxycarbonyl)-1,4,7-triazacyclononane (**25a**)

To a solution of 4-(bromomethyl)phenylboronic acid **24a**(34) (66.1 mg, 0.308 mmol, 1.2 equiv) in MeCN (2.5 mL), 2Boc-tacn **23**(33) (84.7 mg, 0.257 mmol) and potassium carbonate (44.0 mg, 0.318 mmol, 1.2 equiv) were added and the resulting mixture was stirred at reflux for 4 h. After adding H_2_O, the reaction mixture was extracted with CHCl_3_. The organic layer was washed with brine, dried over Na_2_SO_4_, and concentrated under reduced pressure. The resulting residue was purified by silica gel column chromatography (CHCl_3_/MeOH = 50/1) to afford **25a** (122.3 mg, 0.264 mmol, quant.) as a colorless amorphous solid: mp 106–110 °C; ^1^H NMR (400 MHz, acetone-*d*_6_, TMS): δ 1.44 (s, 9H), 1.51 (d, *J* = 5.2 Hz, 9H), 2.64–2.72 (m, 4H), 3.18–3.31 (m, 4H), 3.47–3.54 (m, 4H), 3.66–3.74 (m, 2H), 7.07–7.10 (m, 2H), 7.39–7.42 (m, 2H), 7.76–7.86 (m, 2H) ppm; ^13^C NMR (100 MHz, acetone-*d*_6_, TMS): δ 27.86, 48.29–49.84 (m), 50.15–51.53 (m), 52.89–54.34 (m), 60.39–60.58 (m), 78.6–78.69 (m), 128.02–128.41 (m), 133.97–134.16 (m), 142.53, 155.02, 155.20 ppm; ^11^B NMR (128 MHz, acetone-*d*_6_, BF_3_·OEt_2_): δ 29.3 (br s) ppm; IR (ATR) ν: 3407, 2974, 1668, 1462, 1410, 1365, 1247, 1143, 999, 856, 752, 648, 532, 491, 458, 436 cm^–1^; HRMS (ESI^+^) *m*/*z*: calcd for [M + H]^+^ C_23_H_39_^10^BN_3_O_6_, 463.2963; found, 463.2976; Anal. Calcd (%) for C_23_H_38_BN_3_O_6_·0.2CHCl_3_: C, 57.19; H, 7.90; N, 8.62. Found: C, 57.32; H, 7.66; N, 8.42.

#### 1-[(3-Boronopheny)methyl]-4,7-bis(*tert*-butoxycarbonyl)-1,4,7-triazacyclononane (**25b**)

To a solution of 3-(bromomethyl)phenylboronic acid **24b**(36) (70.1 mg, 0.326 mmol, 1.2 equiv) in MeCN (2.5 mL), 2Boc-tacn **23**(33) (90.1 mg, 0.273 mmol) and potassium carbonate (46.0 mg, 0.333 mmol, 1.2 equiv) were added and the mixture was stirred at reflux for 26 h. After adding H_2_O, the reaction mixture was extracted with CHCl_3_. The organic layer was washed with brine, dried over Na_2_SO_4_, and concentrated under reduced pressure. The resulting residue was purified by silica gel column chromatography (hexanes/AcOEt = 1/1 to CHCl_3_/MeOH = 50/1) to afford **25b** (120.8 mg, 0.261 mmol, 95%) as a colorless amorphous solid: mp 95–97 °C; ^1^H NMR (400 MHz, acetone-*d*_6_, TMS): δ 1.43 (*J* = 3.6 Hz, 9H), 1.50 (*J* = 3.6 Hz, 9H), 2.67–2.74 (m, 4H), 3.21–3.26 (m, 4H), 3.47–3.51 (m, 4H), 3.66–3.76 (m, 2H), 7.13 (s, 2H), 7.26–7.34 (m, 1H), 7.49–7.57 (m, 1H), 7.73–7.86 (m, 2H) ppm; ^13^C NMR (100 MHz, acetone-*d*_6_, TMS): δ 28.70, 49.38–50.24 (m), 50.70–51.33 (m), 52.18, 53.75–53.98 (m), 55.09–55.25 (m), 61.89–61.98 (m), 79.54–79.68 (m), 128.11–128.19 (m), 131.96–132.13 (m), 133.62, 135.77–135.90 (m), 139.92, 155.88–156.13 (m) ppm; ^11^B NMR (128 MHz, acetone-*d*_6_, BF_3_·OEt_2_): δ 29.0 (br s) ppm; IR (ATR) ν: 3407, 2974, 2931, 1669, 1460, 1413, 1364, 1246, 1142, 1093, 997, 856, 751, 710, 665, 621, 527, 459, 436 cm^–1^; HRMS (ESI^+^) *m*/*z*: calcd for [M + H]^+^ C_23_H_39_^10^BN_3_O_6_, 463.2968; found, 463.2963; Anal. Calcd (%) for C_23_H_38_BN_3_O_6_: C, 59.62; H, 8.27; N, 9.07. Found: C, 59.93; H, 8.24; N, 8.81.

#### 1-[(4-Boronopheny)methyl]-1,4,7-triazacyclononane TFA Salt (2TFA) (**12a**)

TFA (1.5 mL) was added to a solution of **25a** (122.3 mg, 0.264 mmol) in CH_2_Cl_2_ (1.5 mL), and the resulting mixture was stirred at room temperature for 1 h. After evaporation, the resulting residue was dissolved in MeCN and reprecipitated with Et_2_O to afford **12a** (95.7 mg, 0.195 mmol, 74%) as colorless powder, which was determined to be the 2TFA salt by elemental analysis: mp 138–141 °C; ^1^H NMR (400 MHz, D_2_O, TSP): δ 3.06 (t, *J* = 5.6 Hz, 4H), 3.24 (t, *J* = 5.6 Hz, 4H), 3.64 (s, 4H), 3.95 (s, 2H), 7.49 (d, *J* = 7.6 Hz, 2H), 7.83 (d, *J* = 7.6 Hz, 2H) ppm; ^13^C NMR (100 MHz, D_2_O, 1,4-dioxane): δ 42.84, 44.32, 48.35, 59.57, 116.93, 130.33, 134.65, 138.81, 163.43 ppm; ^11^B NMR (128 MHz, D_2_O, BF_3_·OEt_2_): δ 29.0 (br s) ppm; IR (ATR) ν: 3005, 2774, 1665, 1610, 1485, 1420, 1397, 1384, 1353, 1183, 1130, 1083, 1055, 1004, 875, 841, 795, 723, 696, 654, 518, 410 cm^–1^; HRMS (ESI^+^) *m*/*z*: calcd for [M + H]^+^ C_13_H_23_^10^BN_3_O_2_, 263.1920; found, 263.1914; Anal. Calcd (%) for C_13_H_22_BN_3_O_2_·2TFA: C, 41.57; H, 4.93; N, 8.55. Found: C, 41.72; H, 4.85; N, 8.54.

#### 1-[(3-Boronopheny)methyl]-1,4,7-triazacyclononane TFA Salt (2TFA) (**12b**)

TFA (1.0 mL) was added to a solution of **25b** (81.4 mg, 0.174 mmol) in CH_2_Cl_2_ (1.0 mL), and the mixture was stirred at room temperature for 1 h. After evaporation, the resulting residue was dissolved in MeCN and reprecipitated with Et_2_O to afford **12b** (60.9 mg, 0.124 mmol, 71%) as colorless powder, which was determined to be the 2TFA salt by elemental analysis: mp 136–138 °C; ^1^H NMR (400 MHz, D_2_O, TSP): δ 3.00 (t, *J* = 6.0 Hz, 4H), 3.15 (br s, 4H), 3.51 (br s, 4H), 3.94 (s, 2H), 7.51 (t, *J* = 8.0 Hz, 1H), 7.57 (d, *J* = 8.0 Hz, 1H), 7.79–7.80 (m, 2H) ppm; ^13^C NMR (100 MHz, D_2_O, 1,4-dioxane): δ 42.84, 44.17, 48.42, 59.82, 129.02, 116.94, 133.21, 133.94, 135.85, 136.01 ppm; ^11^B NMR (128 MHz, D_2_O, BF_3_·OEt_2_): δ 29.3 (br s) ppm; IR (ATR) ν: 2810, 1667, 1429, 1337, 1180, 1126, 1010, 834, 797, 719, 582, 515, 441, 414 cm^–1^; HRMS (ESI^+^) *m*/*z*: calcd for [M + H]^+^ C_13_H_23_^10^BN_3_O_2_, 263.1920; found, 263.1914; Anal. Calcd (%) for C_13_H_22_BN_3_O_2_·2TFA–0.8H_2_O: C, 42.83; H, 4.74; N, 8.81. Found: C, 42.88; H, 5.04; N, 8.81.

#### 1-[4-(13,15-Dioxa-15-boradispiro[5.0.5.3]pentadec-14-yl)phenyl]methyl-1,4,7-triazacyclononane (**15a**)

A mixture of **12a** (30.0 mg, 0.0611 mmol) and bicyclohexyl-1,1′-diol **26**(35) (15.7 mg, 0.0792 mmol, 1.3 equiv) in EtOH (0.8 mL) was refluxed for 6 h. After evaporation, the resulting residue was purified by NH silica gel column chromatography (CHCl_3_/MeOH = 20/1) to afford **15a** (24.8 mg, 0.0583 mmol, 95%) as a colorless amorphous solid: mp 65–67 °C; ^1^H NMR (400 MHz, CDCl_3_, TMS): δ 1.13–1.32 (m, 6H), 1.63–1.81 (m, 14H), 2.61–2.67 (m, 8H), 2.78 (s, 4H), 3.73 (s, 2H), 7.34 (d, *J* = 8.0 Hz, 2H), 7.82 (d, *J* = 7.6 Hz, 2H) ppm; ^13^C NMR (100 MHz, CDCl_3_, TMS): δ 22.32, 25.81, 32.47, 46.40, 46.54, 52.76, 61.71, 84.64, 128.33, 134.92, 142.61 ppm; ^11^B NMR (128 MHz, CDCl_3_, BF_3_·OEt_2_): δ 31.5 (br s) ppm; IR (ATR) ν: 2929, 2851, 1611, 1449, 1398, 1356, 1284, 1238, 1131, 1087, 1018, 937, 822, 750, 652, 506, 418 cm^–1^; HRMS (ESI^+^) *m*/*z*: calcd for [M + H]^+^ C_25_H_41_^10^BN_3_O_2_, 425.3328; found, 425.3322; Anal. Calcd (%) for C_25_H_40_BN_3_O_2_·0.2CHCl_3_·0.6MeOH: C, 66.14; H, 9.17; N, 8.97. Found: C, 66.05; H, 8.98; N, 8.70.

#### 1-[3-(13,15-Dioxa-15-boradispiro[5.0.5.3]pentadec-14-yl)phenyl]methyl-1,4,7-triazacyclononane (**15b**)

A mixture of **12b** (20.0 mg, 0.041 mmol) and bicyclohexyl-1,1′-diol **26**(35) (10.5 mg, 0.053 mmol, 1.3 equiv) in EtOH (0.5 mL) was refluxed for 6 h. After evaporation, the resulting residue was purified by NH silica gel column chromatography (CHCl_3_/MeOH = 20/1) to afford **15b** (11.8 mg, 0.028 mmol, 68%) as a colorless amorphous solid: mp 57–58 °C; ^1^H NMR (400 MHz, CDCl_3_, TMS): δ 1.14–1.33 (m, 6H), 1.63–1.84 (m, 14H), 2.65–2.69 (m, 8H), 2.83 (s, 4H), 3.74 (s, 2H), 7.34 (t, *J* = 7.6 Hz 1H), 7.46 (d, *J* = 8.0 Hz 1H), 7.75 (d, *J* = 7.6 Hz 1H), 7.79 (s, 1H) ppm; ^13^C NMR (100 MHz, CDCl_3_, TMS): δ 22.34, 25.81, 32.48, 46.58, 46.86, 52.95, 61.54, 84.71, 127.75, 131,66, 133.64, 135.24, 138.84 ppm; ^11^B NMR (128 MHz, CDCl_3_, BF_3_·OEt_2_): δ 30.8 (br s) ppm; IR (ATR) ν: 2928, 2852, 1448, 1353, 1285, 1239, 1200, 1145, 1131, 1076, 1040, 939, 779, 751, 708, 615, 507, 409 cm^–1^; HRMS (ESI^+^) *m*/*z*: calcd for [M + H]^+^ C_25_H_41_^10^BN_3_O_2_, 425.3323; found, 425.3327; Anal. Calcd (%) for C_25_H_40_BN_3_O_2_·0.1CHCl_3_·MeOH: C, 66.78; H, 9.47; N, 8.95. Found: C, 66.88; H, 9.29; N, 8.57.

#### 1-[(4-Boronopheny)methyl]-4,7,10-tris(*tert*-butoxycarbonyl)-1,4,7,10-tetraazacyclododecane (**28a**)

To a solution of 4-(bromomethyl)phenylboronic acid **24a**(34) (66.4 mg, 0.309 mmol, 1.5 equiv) in MeCN (2.0 mL), 3Boc-cyclen **27**(38) (100 mg, 0.212 mmol) and potassium carbonate (58.1 mg, 0.420 mmol, 2.0 equiv) were added and the mixture was stirred at reflux for 3 h. After adding H_2_O, the reaction mixture was extracted with CHCl_3_. The organic layer was washed with brine, dried over Na_2_SO_4_, and concentrated under reduced pressure. The resulting residue was purified by silica gel column chromatography (CHCl_3_/MeOH = 100/1) to afford **28a** (108.3 mg, 0.179 mmol, 84%) as a colorless amorphous solid: mp 112–115 °C; ^1^H NMR (300 MHz, CDCl_3_, TMS): δ 1.44–1.49 (m, 27H), 2.71 (br s, 4H), 3.30–3.40 (m, 8H), 3.59 (br s, 4H), 3.70–3.74 (m, 2H), 7.29 (d, *J* = 8.0 Hz, 1H), 7.40 (d, *J* = 8.0 Hz, 1H), 7.70 (d, *J* = 8.0 Hz, 1H), 8.18 (d, *J* = 7.6 Hz, 1H) ppm; ^13^C NMR (100 MHz, CDCl_3_, TMS): δ 18.45, 28.49, 28.72, 2872, 29.70, 47.39, 49.82, 58.50, 79.68, 129.46, 129.97, 133.59, 135.61, 139.38, 155.34, 156.18, 158.78, 163.43, 196.70, 209.38, 210.84 ppm; ^11^B NMR (128 MHz, CDCl_3_, BF_3_·OEt_2_): δ 29.1 (br s) ppm; IR (ATR) ν: 3397, 2975, 2931, 1682, 1668, 1611, 1478, 1457, 1412, 1364, 1341, 1249, 1151, 1109, 1019, 979, 856, 770, 754, 734, 649, 555, 516, 458 cm^–1^; HRMS (ESI^+^) *m*/*z*: calcd for [M + H]^+^ C_30_H_52_^10^BN_4_O_8_, 606.3909; found, 606.3917; Anal. Calcd (%) for C_30_H_51_BN_4_O_8_·0.3CHCl_3_: C, 56.65; H, 8.05; N, 8.72. Found: C, 56.73; H, 8.10; N, 8.66.

#### 1-[(3-Boronopheny)methyl]-4,7,10-tris(*tert*-butoxycarbonyl)-1,4,7,10-tetraazacyclododecane (**28b**)^20^

To a solution of 3-(bromomethyl)phenylboronic acid **24b**(36) (67.8 mg, 0.316 mmol, 1.5 equiv) in MeCN (2.0 mL), 3Boc-cyclen **27**(38) (100 mg, 0.212 mmol) and potassium carbonate (58.4 mg, 0.423 mmol, 2.0 equiv) were added and the mixture was stirred at reflux for 3 h. After adding H_2_O, the reaction mixture was extracted with CHCl_3_. The organic layer was washed with brine, dried over Na_2_SO_4_, and concentrated under reduced pressure. The resulting residue was purified by silica gel column chromatography (CHCl_3_/MeOH = 100/1) to afford **28b** (121.8 mg, 0.201 mmol, 95%) as a colorless amorphous solid; the ^1^H and ^13^C and ^11^B NMR spectra of product were identical to previously reported data.^20^

#### 1-[(4-Boronopheny)methyl]-1,4,7,10-tetraazacyclododecane TFA Salt (2TFA) (**13a**)

TFA (1.0 mL) was added to a solution of **28a** (94.2 mg, 0.155 mmol) in CH_2_Cl_2_ (1.0 mL), and the mixture was stirred at room temperature for 1 h. After evaporation, the resulting residue was dissolved in AcOEt and reprecipitated with hexanes to afford **13a** (72.5 mg, 0.136 mmol, 88%) as colorless powder, which were determined to be the 2TFA salt by elemental analysis: mp 172–175 °C; ^1^H NMR (400 MHz, D_2_O, TSP): δ 2.93–3.01 (m, 8H), 3.17–3.25 (m, 8H), 3.88 (s, 2H), 7.44 (d, *J* = 8.0 Hz, 2H), 7.83 (d, *J* = 8.0 Hz, 2H) ppm; ^13^C NMR (100 MHz, D_2_O, 1,4-dioxane): δ 42.31, 42.46, 44.78, 48.38, 57.18, 129.96, 134.87, 138.74 ppm; ^11^B NMR (128 MHz, D_2_O, BF_3_·OEt_2_): δ 29.05 (br s) ppm; IR (ATR) ν: 3301, 3088, 2856, 1668, 1610, 1454, 1409, 1343, 1196, 1176, 1120, 1052, 1017, 829, 796, 719, 693, 651, 596, 516, 435, 414 cm^–1^; HRMS (ESI^+^) *m*/*z*: calcd for [M + H]^+^ C_15_H_28_^10^BN_4_O_2_, 306.2342; found, 306.2336; Anal. Calcd (%) for C_15_H_27_BN_4_O_2_·2TFA: C, 42.71; H, 5.47; N, 10.49. Found: C, 42.75; H, 5.38; N, 10.44.

#### 1-[(3-Boronopheny)methyl]-1,4,7,10-tetraazacyclododecane (**13b**)^20^

TFA (2.0 mL) was added to a solution of **28b** (110.2 mg, 0.182 mmol) in CH_2_Cl_2_ (2.0 mL), and the mixture was stirred at room temperature for 1 h. After evaporation, the resulting residue was purified by NH silica gel column chromatography (CHCl_3_/MeOH = 20/1) to afford **13b** (50.4 mg, 0.165 mmol, 91%) as a colorless amorphous solid. The ^1^H, ^13^C and ^11^B NMR spectra of product were identical to previously reported data.^20^

#### 1-[4-(13,15-Dioxa-15-boradispiro[5.0.5.3]pentadec-14-yl)phenyl]methyl-1,4,7,10-tetraazacyclododecane (**16a**)

A mixture of **13a** (40.0 mg, 0.0748 mmol) and bicyclohexyl-1,1′-diol **26**(35) (15.2 mg, 0.0766 mmol, 1.0 equiv) in EtOH (1.0 mL) was refluxed for 6 h. After evaporation, the resulting residue was dissolved in EtOH and reprecipitated with hexanes, and the resulting precipitate was purified by NH silica gel column chromatography (CHCl_3_/MeOH = 20/1) to afford **16a** (34.5 mg, 0.0736 mmol, 98%) as a colorless solid: mp 129–131 °C; ^1^H NMR (300 MHz, CDCl_3_, TMS): δ 1.13–1.31 (m, 6H), 1.62–1.83 (m, 14H), 2.57 (t, *J* = 5.2 Hz, 8H), 2.67 (t, *J* = 5.2 Hz, 4H), 2.81 (t, *J* = 5.6 Hz, 4H), 3.63 (s, 2H), 7.31 (d, *J* = 8.0 Hz, 2H), 7.81 (d, *J* = 8.0 Hz, 2H) ppm; ^13^C NMR (100 MHz, CDCl_3_, TMS): δ 22.31, 25.81, 32.45, 45.09, 46.34, 47.14, 51.26, 59.33, 84.51, 128.36, 134.95, 141.93 ppm; ^11^B NMR (128 MHz, CDCl_3_, BF_3_·OEt_2_): δ 31.1 (br s) ppm; IR (ATR) ν: 2933, 2856, 2811, 1610, 1450, 1403, 1356, 1319, 1284, 1272, 1239, 1131, 1088, 1041, 1020, 937, 911, 822, 801, 746, 725, 653, 540, 507 cm^–1^; HRMS (ESI^+^) *m*/*z*: calcd for [M + H]^+^ C_27_H_46_^10^BN_4_O_2_, 468.3750; found, 468.3745; Anal. Calcd (%) for C_27_H_45_BN_4_O_2_·0.5MeOH: C, 68.17; H, 9.78; N, 11.56. Found: C, 68.35; H, 9.79; N, 11.26.

#### 1-[3-(13,15-Dioxa-15-boradispiro[5.0.5.3]pentadec-14-yl)phenyl]methyl-1,4,7,10-tetraazacyclododecane (**16b**)

A mixture of **13b** (27.0 mg, 0.0882 mmol) and bicyclohexyl-1,1′-diol **26**(35) (17.5 mg, 0.0882 mmol, 1.0 equiv) in EtOH (0.9 mL) was refluxed for 3 h. After evaporation, the resulting residue was purified by NH silica gel column chromatography (CHCl_3_/MeOH = 20/1) to afford **16b** (33.5 mg, 0.0714 mmol, 81%) as a colorless amorphous solid: mp 46–48 °C; ^1^H NMR (400 MHz, CDCl_3_, TMS): δ 1.22–1.32 (m, 6H), 1.63–1.83 (m, 14H), 2.56–2.59 (m, 8H), 2.67 (t, *J* = 4.4 Hz, 4H), 2.81 (t, *J* = 4.4 Hz, 4H), 3.64 (s, 2H), 7.32 (t, *J* = 7.6 Hz, 1H), 7.41 (d, *J* = 8.0 Hz, 1H), 7.74 (d, *J* = 7.2 Hz, 1H), 7.77 (s, 1H) ppm; ^13^C NMR (100 MHz, CDCl_3_, TMS): δ 22.31, 25.80, 32.45, 45.34, 46.40, 47.39, 51.38, 59.26, 84.57, 127.71, 131.79, 133.80, 135.43, 138.04 ppm; ^11^B NMR (128 MHz, CDCl_3_, BF_3_·OEt_2_): δ 30.8 (br s) ppm; IR (ATR) ν: 2929, 2851, 1604, 1448, 1429, 1392, 1352, 1320, 1284, 1272, 1253, 1239, 1200, 1146, 1131, 1114, 1077, 1039, 939, 909, 834, 803, 747, 707, 681, 658, 541, 507 cm^–1^; HRMS (ESI^+^) *m*/*z*: calcd for [M + H]^+^ C_27_H_46_^10^BN_4_O_2_, 468.3750; found, 468.3745; Anal. Calcd (%) for C_27_H_45_BN_4_O_2_·0.1CHCl_3_·2.4MeOH: C, 63.58; H, 9.89; N, 10.05. Found: C, 63.90; H, 9.63; N, 9.65.

#### 1-[(4-Boronopheny)methyl]-4,7,10,13-tetra(*tert*-butoxycarbonyl)-1,4,7,10,13-pentaazacyclopentadecane (**30a**)

To a solution of 4-(bromomethyl)phenylboronic acid **24a**(34) (48.3 mg, 0.225 mmol, 1.2 equiv) in MeCN (1.5 mL), **29**(39) (113.9 mg, 0.185 mmol) and potassium carbonate (38.1 mg, 0.276 mmol, 1.5 equiv) were added and the mixture was stirred at reflux for 2 h. After adding H_2_O, the reaction mixture was extracted with CHCl_3_. The organic layer was washed with brine, dried over Na_2_SO_4_, and concentrated under reduced pressure. The resulting residue was purified by silica gel column chromatography (CHCl_3_/MeOH = 100/1) to afford **30a** (115.0 mg, 0.153 mmol, 83%) as a colorless amorphous solid: mp 94–96 °C; ^1^H NMR (400 MHz, acetone-*d*_6_, TMS): δ 1.30 (s, 9H), 1.44 (s, 9H), 1.48 (s, 18H), 2.74 (s, 4H), 3.47 (s, 16H), 3.67 (s, 2H), 7.09 (s, 2H), 7.34 (d, *J* = 8.0 Hz, 2H), 7.83 (d, *J* = 8.0 Hz, 2H) ppm; ^13^C NMR (100 MHz, acetone-*d*_6_, TMS): δ 28.63, 31.99, 47.35, 53.23, 55.49, 60.21, 69.71, 79.56, 79.89, 128.59, 135.05, 142.72, 155.59, 155.72 ppm; ^11^B NMR (128 MHz, acetone-*d*_6_, BF_3_·OEt_2_): δ 29.7 (br s) ppm; IR (ATR) ν: 3420, 2976, 1682, 1465, 1411, 1365, 1245, 1155, 1018, 858, 753, 648, 559, 458 cm^–1^; HRMS (ESI^+^) *m*/*z*: calcd for [M + H]^+^ C_37_H_65_^10^BN_5_O_10_, 749.4861; found, 749.4855; Anal. Calcd (%) for C_37_H_64_BN_5_O_10_ 0.3H_2_O: C, 58.85; H, 8.62; N, 9.27. Found: C, 58.88; H, 8.56; N, 9.06.

#### 1-[(3-Boronopheny)methyl]-4,7,10,13-tetra(*tert*-butoxycarbonyl)-1,4,7,10,13-pentaazacyclopentadecane (**30b**)

To a solution of 3-(bromomethyl)phenylboronic acid **24b**(36) (51.6 mg, 0.240 mmol, 1.2 equiv) in MeCN (2.0 mL), **29**(39) (121.0 mg, 0.196 mmol) and potassium carbonate (40.6 mg, 0.294 mmol, 1.5 equiv) were added and the mixture was stirred at reflux for 4 h. After adding H_2_O, the reaction mixture was extracted with CHCl_3_. The organic layer was washed with brine, dried over Na_2_SO_4_, and concentrated under reduced pressure. The resulting residue was purified by silica gel column chromatography (CHCl_3_/MeOH = 100/1) to afford **30b** (127.4 mg, 0.170 mmol, 87%) as a colorless amorphous solid: mp 106–110 °C; ^1^H NMR (400 MHz, acetone-*d*_6_, TMS): δ 1.29 (s, 9H), 1.45 (s, 9H), 1.47 (s, 18H), 2.74 (s, 4H), 3.47 (s, 16H), 3.68 (s, 2H), 7.15 (s, 2H), 7.29 (t, *J* = 7.2 Hz, 1H), 7.40 (d, *J* = 6.8 Hz, 1H), 7.72 (d, *J* = 5.6 Hz, 1H), 7.82 (s, 1H) ppm; ^13^C NMR (100 MHz, acetone-*d*_6_, TMS): δ 28.61, 31.99, 46.54, 47.34, 53.22, 55.44, 60.19, 79.65, 79.89, 128.26, 131.44, 133.58, 135.15, 155.74 ppm; ^11^B NMR (128 MHz, acetone-*d*_6_, BF_3_·OEt_2_): δ 30.0 (br s) ppm; IR (ATR) ν: 3419, 2976, 1682, 1464, 1413, 1365, 1244, 1155, 1043, 860, 770, 710, 558, 462, 418 cm^–1^; HRMS (ESI^+^) *m*/*z*: calcd for [M + H]^+^ C_37_H_65_^10^BN_5_O_10_, 749.4861; found, 749.4855; Anal. Calcd (%) for C_37_H_64_BN_5_O_10_ 0.25CHCl_3_: C, 57.39; H, 8.31; N, 8.92. Found: C, 57.67; H, 8.21; N, 8.72.

#### 1-[(2-Boronopheny)methyl]-4,7,10,13-tetra(*tert*-butoxycarbonyl)-1,4,7,10,13-pentaazacyclopentadecane (**30c**)

To a solution of 2-(bromomethyl)phenylboronic acid **24c**(37) (55.1 mg, 0.256 mmol, 1.2 equiv) in MeCN (2.0 mL), **29**(39) (129.8 mg, 0.211 mmol) and potassium carbonate (44.3 mg, 0.32 mmol, 1.5 equiv) were added and the mixture was stirred at reflux for 10 h. After adding H_2_O, the reaction mixture was extracted with CHCl_3_. The organic layer was washed with brine, dried over Na_2_SO_4_, and concentrated under reduced pressure. The resulting residue was purified by silica gel column chromatography (CHCl_3_/MeOH = 100/1) to afford **30c** (115.7 mg, 0.154 mmol, 73%) as a colorless amorphous solid: mp 105–109 °C; ^1^H NMR (400 MHz, acetone-*d*_6_, TMS): δ 1.47 (s, 36H), 2.82 (s, 4H), 3.45 (s, 16H), 3.82 (s, 2H), 7.25–7.36 (m, 4H), 7.87 (br, 1H), 8.78 (br, 1H) ppm; ^13^C NMR (100 MHz, acetone-*d*_6_, TMS): δ 28.39, 28.63, 47.12, 50.94, 51.42, 55.49, 62.29, 79.77, 79.94, 127.94, 130.61, 131.96, 137.26, 142.60, 155.44, 155.62 ppm; ^11^B NMR (128 MHz, acetone-*d*_6_, BF_3_·OEt_2_): δ 29.7 (br s) ppm; IR (ATR) ν: 2975, 2932, 1692, 1463, 1412, 1391, 1365, 1309, 1244, 1156, 1032, 947, 894, 860, 770, 653, 550, 460 cm^–1^; HRMS (ESI^+^) *m*/*z*: calcd for [M + H]^+^ C_37_H_65_^10^BN_5_O_10_, 749.4861; found, 749.4864; Anal. Calcd (%) for C_37_H_64_BN_5_O_10_ 1.5MeOH: C, 57.96; H, 8.84; N, 8.78. Found: C, 57.84; H, 9.06; N, 9.07.

#### 1-[(4-Boronopheny)methyl]-1,4,7,10,13-pentaazacyclopentadecane TFA Salt (3TFA) (**14a**)

TFA (1.5 mL) was added to a solution of **30a** (111.0 mg, 0.148 mmol) in CH_2_Cl_2_ (1.5 mL), and the mixture was stirred at room temperature for 30 min. After evaporation, the resulting residue was dissolved in MeCN and reprecipitated with Et_2_O to afford **14a** (90.0 mg, 0.130 mmol, 88%) as colorless powder, which was determined to be the 3TFA salt by elemental analysis: mp 136–137 °C; ^1^H NMR (400 MHz, D_2_O, TSP): δ 3.01 (t, *J* = 6.0 Hz, 4H), 3.17 (s, 4H), 3.26–3.33 (m, 8H), 3.38 (t, *J* = 6.0 Hz, 4H), 3.95 (s, 2H), 7.43 (d, *J* = 8.0 Hz, 2H), 7.83 (d, *J* = 8.0 Hz, 2H) ppm; ^13^C NMR (100 MHz, D_2_O, 1,4-dioxane): δ 44.31, 45.30, 45.81, 50.24, 56.73, 116.92, 130.56, 134.66, 137.03, 163.59 ppm; ^11^B NMR (128 MHz, D_2_O, BF_3_·OEt_2_): δ 29.2 (br s) ppm; IR (ATR) ν: 3029, 1668, 1410, 1382, 1359, 1178, 1129, 1055, 1018, 888, 838, 798, 720, 698, 654, 517 cm^–1^; HRMS (ESI^+^) *m*/*z*: calcd for [M + H]^+^ C_17_H_33_^10^BN_5_O_2_, 349.2764; found, 349.2770; Anal. Calcd (%) for C_17_H_32_BN_5_O_2_·3TFA: C, 39.96; H, 5.10; N, 10.13. Found: C, 39.97; H, 4.98; N, 10.05.

#### 1-[(3-Boronopheny)methyl]-1,4,7,10,13-pentaazacyclopentadecane TFA Salt (3TFA) (**14b**)

TFA (1.5 mL) was added to a solution of **30b** (127.2 mg, 0.170 mmol) in CH_2_Cl_2_ (1.5 mL), and the mixture was stirred at room temperature for 1 h. After evaporation, the resulting residue was dissolved in MeCN and reprecipitated with Et_2_O to afford **14b** (107.1 mg, 0.155 mmol, 91%) as colorless powder, which was determined to be the 3TFA salt by elemental analysis: mp 106–108 °C; ^1^H NMR (400 MHz, D_2_O, TSP): δ 3.03 (t, *J* = 5.2 Hz 4H), 3.18 (s, 4H), 3.27–3.29 (m, 4H), 3.33 (t, *J* = 5.2 Hz 4H), 3.37–3.40 (m, 4H), 3.98 (s, 2H), 7.48–7.55 (m, 2H), 7.73 (s, 1H), 7.80–7.83 (m, 1H) ppm; ^13^C NMR (100 MHz, D_2_O, 1,4-dioxane): δ 44.34, 45.21, 45.49, 45.79, 50.27, 56.94, 116.92, 129.09, 133.53, 133.62, 134.35, 136.20, 163.58 ppm; ^11^B NMR (128 MHz, D_2_O, BF_3_·OEt_2_): δ 28.6 (br s) ppm; IR (ATR) ν: 3030, 2856, 1666, 1426, 1337, 1179, 1123, 834, 797, 719, 597, 517 cm^–1^; HRMS (ESI^+^) *m*/*z*: calcd for [M + H]^+^ C_17_H_33_^10^BN_5_O_2_, 349.2764; found, 349.2758; Anal. Calcd (%) for C_17_H_32_BN_5_O_2_·3.4TFA: C, 38.79; H, 4.82; N, 9.43. Found: C, 38.66; H, 4.95; N, 9.33.

#### 1-[(2-Boronopheny)methyl]-1,4,7,10,13-pentaazacyclopentadecane TFA Salt (3TFA) (**14c**)

TFA (1.5 mL) was added to a solution of **30c** (115.7 mg, 0.154 mmol) in CH_2_Cl_2_ (1.5 mL), and the mixture was stirred at room temperature for 1 h. After evaporation, the resulting residue was dissolved in AcOEt and reprecipitated with hexanes to afford **14c** (94.8 mg, 0.137 mmol, 89%) as colorless powder, which was determined to be the 3TFA salt by elemental analysis: mp 118–120 °C; ^1^H NMR (400 MHz, D_2_O, TSP): δ 2.99 (t, *J* = 4.8 Hz 4H), 3.11 (m, *J* = 5.6 Hz 4H), 3.17 (s, 12H), 4.02 (s, 2H), 7.40 (d, *J* = 6.8 Hz 1H), 7.47–7.51 (m, 2H), 7.70 (d, *J* = 7.2 Hz 1H) ppm; ^13^C NMR (100 MHz, D_2_O, 1,4-dioxane): δ 44.60, 44.86, 45.50, 51.06, 60.39, 116.91, 128.88, 130.75, 131.36, 134.06, 138.21, 163.60 ppm; ^11^B NMR (128 MHz, D_2_O, BF_3_·OEt_2_): δ 29.7 (br s) ppm; IR (ATR) ν: 3023, 2843, 1668, 1440, 1359, 1193, 1147, 1126, 1050, 1032, 836, 797, 767, 719, 624, 588, 517, 443, 420 cm^–1^; HRMS (ESI^+^) *m*/*z*: calcd for [M + H]^+^ C_17_H_33_^10^BN_5_O_2_, 349.2764; found, 349.2769; Anal. Calcd (%) for C_17_H_32_BN_5_O_2_·3TFA: C, 39.96; H, 5.10; N, 10.13. Found: C, 39.67; H, 4.87; N, 9.84.

#### 1-[4-(13,15-Dioxa-15-boradispiro[5.0.5.3]pentadec-14-yl)phenyl]methyl-1,4,7,10,13-pentaazacyclopentadecane (**17a**)

A mixture of **14a** (30.4 mg, 0.044 mmol) and bicyclohexyl-1,1′-diol **26**(35) (9.4 mg, 0.0474 mmol, 1.1 equiv) in EtOH (0.8 mL) was refluxed for 2 h. After evaporation, the resulting residue was purified by NH silica gel column chromatography (CHCl_3_/MeOH = 100/1) to afford **17a** (21.4 mg, 0.0419 mmol, 88%) as a colorless amorphous solid: mp 43–44 °C; ^1^H NMR (400 MHz, CDCl_3_, TMS): δ 1.16–1.31 (m, 6H), 1.73–1.80 (m, 14H), 2.65 (s, 12H), 2.79 (s, 8H), 3.62 (s, 2H), 7.32 (d, *J* = 8.0 Hz, 2H), 7.81 (d, *J* = 7.6 Hz, 2H) ppm; ^13^C NMR (100 MHz, CDCl_3_, TMS): δ 22.31, 25.81, 32.47, 47.29, 47.79, 48.24, 49.00, 54.55, 59.62, 84.68, 128.36, 134.93, 142.52 ppm; ^11^B NMR (128 MHz, CDCl_3_, BF_3_·OEt_2_): δ 30.3 (br s) ppm; IR (ATR) ν: 3287, 2930, 2849, 1610, 1449, 1399, 1356, 1284, 1238, 1130, 1087, 937, 823, 727, 652, 507 cm^–1^; HRMS (ESI^+^) *m*/*z*: calcd for [M + Na]^+^, C_29_H_50_^10^BN_5_O_2_Na, 533.3986; found, 533.4010; Anal. Calcd (%) for C_29_H_50_BN_5_O_2_·CHCl_3_: C, 57.11; H, 8.15; N, 11.10. Found: C, 57.46; H, 8.13; N, 10.79.

#### 1-[3-(13,15-Dioxa-15-boradispiro[5.0.5.3]pentadec-14-yl)phenyl]methyl-1,4,7,10,13-pentaazacyclopentadecane (**17b**)

A mixture of **14b** (30.2 mg, 0.0437 mmol) and bicyclohexyl-1,1′-diol **26**(35) (8.7 mg, 0.0439 mmol, 1.0 equiv) in EtOH (0.6 mL) was refluxed for 5 h. After evaporation, the resulting residue was purified by NH silica gel column chromatography (CHCl_3_/MeOH = 20/1) to afford **17b** (15.6 mg, 0.0305 mmol, 70%) as a colorless amorphous solid: ^1^H NMR (400 MHz, CDCl_3_, TMS): δ 1.17–1.33 (m, 6H), 1.76–1.80 (m, 14H), 2.62–2.80 (m, 20H), 3.60 (s, 2H), 7.33 (t, *J* = 7.6 Hz, 1H), 7.47 (d, *J* = 8.0 Hz, 1H), 7.68 (s, 1H), 7.76 (d, *J* = 7.6 Hz, 1H) ppm; ^13^C NMR (100 MHz, CDCl_3_, TMS): δ 22.33, 25.78, 32.48, 47.30, 47.85, 48.33, 48.96, 54.84, 59.63, 84.79, 127.70, 132.04, 133.90, 135.45, 138.83 ppm; ^11^B NMR (128 MHz, CDCl_3_, BF_3_·OEt_2_): δ 30.2 (br s) ppm; IR (ATR) ν: 3281, 2929, 2849, 1550, 1449, 1353, 1272, 1238, 1201, 1130, 1077, 1041, 939, 910, 807, 760, 708, 611, 540, 509 cm^–1^; HRMS (ESI^+^) *m*/*z*: calcd for [M + Na]^+^, C_29_H_50_^10^BN_5_O_2_Na, 533.3986; found, 533.4010; Anal. Calcd (%) for C_29_H_50_BN_5_O_2_: C, 68.09; H, 9.85; N, 13.69. Found: C, 68.06; H, 10.15; N, 13.77.

#### 1-[2-(13,15-Dioxa-15-boradispiro[5.0.5.3]pentadec-14-yl)phenyl]methyl-1,4,7,10,13-pentaazacyclopentadecane (**17c**)

A mixture of **14c** (26.5 mg, 0.0383 mmol) and bicyclohexyl-1,1′-diol **26**(35) (7.6 mg, 0.0383 mmol, 1.0 equiv) in EtOH (0.4 mL) was refluxed for 6 h. After evaporation, the resulting residue was purified by NH silica gel column chromatography (CHCl_3_/MeOH = 30/1) to afford **17c** (15 mg, 0.0293 mmol, 77%) as a colorless solid: mp 78–80 °C; ^1^H NMR (400 MHz, CDCl_3_, TMS): δ 1.14–1.33 (m, 6H), 1.64–1.81 (m, 14H), 2.58–2.77 (m, 20H), 3.94 (s, 2H), 7.23 (t, *J* = 7.2 Hz, 1H), 7.42 (td, *J* = 7.6, 1.2 Hz, 1H), 7.59 (d, *J* = 7.2 Hz, 1H), 7.82 (dd, *J* = 7.2, 1.6 Hz, 1H) ppm; ^13^C NMR (100 MHz, CDCl_3_, TMS): δ 22.52, 25.78, 32.48, 47.57, 48.03, 48.52, 49.16, 55.40, 57.57, 84.76, 125.97, 129.34, 130.78, 135.91, 147.03 ppm; ^11^B NMR (128 MHz, CDCl_3_, BF_3_·OEt_2_): δ 30.4 (br s) ppm; IR (ATR) ν: 3285, 2932, 2814, 1598, 1568, 1439, 1345, 1311, 1284, 1272, 1236, 1132, 1110, 1064, 1039, 938, 805, 749, 733, 655, 545, 507 cm^–1^; HRMS (ESI^+^) *m*/*z*: calcd for [M + H]^+^ C_29_H_51_^10^BN_5_O_2_, 511.4167; found, 511.4173; Anal. Calcd (%) for C_29_H_50_BN_5_O_2_·0.25CHCl_3_: C, 64.89; H, 9.36; N, 12.94. Found: C, 65.04; H, 9.60; N, 12.82.

#### Complexation of **16a** with Zn^2+^ (**19a**)

To a solution of **16a** (10.8 mg, 0.023 mmol) in EtOH (0.3 mL), Zn(NO_3_)_2_·6H_2_O (6.9 mg, 0.0232 mmol, 1.0 equiv) in EtOH (0.2 mL) was added at room temperature. After evaporation, the resulting residue was recrystallized from EtOH (0.1 mL) to provide colorless crystals of **19a** (10.1 mg, 0.015 mmol, 67%): mp 257–259 °C; ^1^H NMR (400 MHz, D_2_O, TSP): δ 1.44–1.83 (m, 20H), 2.74 (br, 2H), 2.87 (br, 8H), 3.00 (br, 4H), 3.26 (br, 2H), 3.83 (br, 1H), 4.04–4.11 (m, 4H), 7.52–7.57 (m, 2H), 7.74–7.92 (m, 2H) ppm; ^13^C NMR (100 MHz, D_2_O, 1,4-dioxane): δ 22.44, 25.51, 32.00, 42.75, 44.17, 45.09, 49.73, 56.24, 87.52, 131.42, 134.44, 135.30 ppm; ^11^B NMR (128 MHz, D_2_O, BF_3_·OEt_2_): δ 29.3 (br s) ppm; IR (ATR) ν: 3243, 2928, 1611, 1482, 1449, 1354, 1284, 1238, 1131, 1088, 992, 935, 856, 819, 730, 702, 673, 644, 538, 473 cm^–1^; HRMS (ESI^+^) *m*/*z*: calcd for [M]^2+^ C_27_H_45_^10^BN_4_O_2_^64^Zn, 265.6476; found, 265.6474; Anal. Calcd (%) for C_27_H_45_BN_6_O_8_Zn·0.5H_2_O: C, 48.63; H, 6.95; N, 12.60. Found: C, 48.58; H, 6.88; N, 12.47.

#### Complexation of **16b** with Zn^2+^ (**19b**)

To a solution of **16b** (15.5 mg, 0.0331 mmol) in EtOH (0.3 mL), Zn(NO_3_)_2_·6H_2_O (9.8 mg, 0.033 mmol, 1.0 equiv) in EtOH (0.2 mL) was added at room temperature. The generated crystalline sample of **19b** was recrystallized from EtOH (0.5 mL) and Et_2_O (0.5 mL) to provide colorless crystals (10.6 mg, 0.016 mmol, 49%): mp 208–210 °C; ^1^H NMR (400 MHz, D_2_O, TSP): δ 1.26 (br, 2H), 1.49 (br, 4H), 1.66–1.81 (m, 14H), 2.73 (br, 2H), 2.86 (br, 8H), 2.99 (br, 4H), 3.24 (br, 2H), 3.84 (br, 1H), 4.05 (s, 2H), 4.10 (br s, 2H), 7.46 (d, *J* = 7.6 Hz, 2H), 7.90 (d, *J* = 7.6 Hz, 2H) ppm; ^13^C NMR (100 MHz, D_2_O, 1,4-dioxane): δ 21.90, 25.84, 30.08, 42.77, 44.17, 45.09, 49.67, 56.33, 77.05, 128.85, 131.42, 134.24, 134.52, 136.86 ppm; ^11^B NMR (128 MHz, D_2_O, BF_3_·OEt_2_): δ 28.3 (br s) ppm; IR (ATR) ν: 3211, 2927, 1495, 1432, 1349, 1283, 1238, 1204, 1131, 1092, 961, 937, 909, 808, 708, 694, 641, 614, 565, 502 cm^–1^; HRMS (ESI^+^) *m*/*z*: calcd for [M]^2+^ C_27_H_45_^10^BN_4_O_2_^64^Zn, 265.6476; found, 265.6475; Anal. Calcd (%) for C_27_H_45_BN_6_O_8_Zn·0.5H_2_O: C, 48.63; H, 6.95; N, 12.60. Found: C, 48.73; H, 7.11; N, 12.37.

### Synthesis of ^10^B-Enriched Compounds

#### 4-(Bromomethyl)phenylboronic Acid (^**10**^**B-24a**)^34^

To a solution of 4-bromotoluene **34a** (904 mg, 5.28 mmol, 1.3 equiv) in THF (10 mL), 1.6 N of *n*-butyllithium (*n*-BuLi) in hexanes (3.3 mL, 5.28 mmol, 1.3 equiv) was added at −78 °C and the reaction mixture was stirred at the same temperature for 1 h, after which, ^10^B-enriched trimethyl borate (>99.5% of ^10^B) (450 μL, 4.06 mmol, 1.0 equiv) was slowly added. After stirring at −78 °C to room temperature overnight, 2 N aqueous HCl was added to the reaction mixture, which was further stirred at 0 °C for 3 h. After extraction with CHCl_3_, the organic layer was washed with brine, dried over Na_2_SO_4_, and concentrated under reduced pressure. The resulting residue was recrystallized from hexanes to afford ^**10**^**B-35a** (279 mg, 2.06 mmol, 51%) as a colorless needle crystal.

A mixture of ^**10**^**B-35a** (270 mg, 2.00 mmol), NBS (390 mg, 2.19 mmol, 1.1 equiv), and benzoyl peroxide (BPO) (16 mg, 0.066 mmol, 0.03 equiv) in CCl_4_ (13 mL) was refluxed for 6 h and then diluted with CHCl_3_. The reaction mixture was washed with H_2_O and brine, dried over Na_2_SO_4_, and evaporated. The resulting residue was recrystallized from hexanes/AcOEt to give ^**10**^**B-24a** (281 mg, 1.31 mmol, 66%) as a colorless solid: isotopic purity of ^10^B: 98.5 ± 0.3%; mp 159–162 °C; ^1^H NMR (400 MHz, DMSO-*d*_6_, TMS): δ 4.69 (s, 2H), 7.40 (d, *J* = 8.4 Hz, 2H), 7.76 (d, *J* = 7.6 Hz, 2H), 8.09 (s, 2H) ppm; ^13^C NMR (100 MHz, DMSO-*d*_6_, TMS): δ 34.53, 125.42, 128.26, 134.39, 139.62 ppm; IR (ATR) ν: 3268, 1613, 1518, 1398, 1373, 1230, 1178, 1110, 1094, 1028, 1014, 844, 803, 744, 691, 659, 633, 601, 500, 442 cm^–1^; HRMS (ESI^+^) *m*/*z*: calcd for [M + Na]^+^ C_7_H_8_^10^BBrO_2_Na, 235.9729; found, 235.9735; Anal. Calcd (%) for C_7_H_8_^10^BBrO_2_·0.1H_2_O: C, 38.95; H, 3.83. Found: C, 38.56; H, 3.52.

#### 3-(Bromomethyl)phenylboronic Acid (^**10**^**B-24b**)^36^

To a solution of 3-bromotoluene **34b** (916 mg, 5.36 mmol, 1.7 equiv) in THF (6 mL), 1.6 N of *n*-BuLi in hexanes (3.5 mL, 5.6 mmol, 1.8 equiv) was added at −78 °C and the reaction mixture was stirred at the same temperature for 1 h, after which, ^10^B-enriched trimethyl borate (>99.5% of ^10^B) (350 μL, 3.16 mmol, 1.0 equiv) was slowly added. After stirring at −78 °C to room temperature overnight, 2 N aqueous HCl was added to the reaction mixture, which was further stirred at 0 °C for 3 h. After extraction with CHCl_3_, the organic layer was washed with brine, dried over Na_2_SO_4_, and concentrated under reduced pressure. The resulting residue was recrystallized from hexanes to afford ^**10**^**B-35b** (103 mg, 0.762 mmol, 24%) as a colorless needle crystal.

A mixture of ^**10**^**B-35b** (98 mg, 0.721 mmol), NBS (140 mg, 0.787 mmol, 1.1 equiv), and BPO (6.0 mg, 0.025 mmol, 0.03 equiv) in CCl_4_ (4 mL) was refluxed for 7 h and then diluted with CHCl_3_. The reaction mixture was washed with H_2_O and brine, dried over Na_2_SO_4_, and evaporated. The resulting residue was recrystallized from hexanes/AcOEt to give ^**10**^**B-24b** (110.2 mg, 0.515 mmol, 71%) as a colorless solid: isotopic purity of ^10^B: 98.8 ± 0.1%; mp 208–211 °C; ^1^H NMR (400 MHz, DMSO-*d*_6_, TMS): δ 4.70 (s, 2H), 7.33 (t, *J* = 8.0 Hz, 1H), 7.48 (d, *J* = 7.6 Hz, 1H), 7.73 (d, *J* = 7.6 Hz, 1H), 7.84 (s, 1H), 8.12 (s, 2H) ppm; ^13^C NMR (100 MHz, DMSO-*d*_6_, TMS): δ 34.90, 127.63, 130.81, 133.92, 134.95, 136.79 ppm; IR (ATR) ν: 3054, 1693, 1604, 1486, 1434, 1370, 1331, 1222, 1200, 1079, 999, 926, 806, 739, 696, 612, 597, 553, 431 cm^–1^; HRMS (ESI^+^) *m*/*z*: calcd for [M + Na]^+^ C_7_H_8_^10^BBrO_2_Na, 235.9729; found, 235.9739; Anal. Calcd (%) for C_7_H_8_^10^BBrO_2_·0.3AcOEt·3H_2_O: C, 43.39; H, 3.35. Found: C, 43.66; H, 3.01.

#### 1-[(3-Boronopheny)methyl]-1,4,7-triazacyclononane TFA Salt (2TFA) (^**10**^**B-12b**)

A mixture of 3-(bromomethyl)phenylboronic acid ^**10**^**B-24b**(36) (35 mg, 0.164 mmol, 1.1 equiv), 2Boc-tacn **23**(33) (50 mg, 0.152 mmol), and potassium carbonate (25.1 mg, 0.182 mmol, 1.2 equiv) in MeCN (1.5 mL) were refluxed for 16 h. After adding H_2_O, the reaction mixture was extracted with CHCl_3_. The organic layer was washed with brine, dried over Na_2_SO_4_, and concentrated under reduced pressure. The resulting residue was purified by silica gel column chromatography (CHCl_3_/MeOH = 50/1) to afford ^**10**^**B-25b** (62.1 mg) as a colorless amorphous solid.

TFA (1.0 mL) was added to a solution of ^**10**^**B-25b** in CH_2_Cl_2_ (1.0 mL), and the mixture was stirred at room temperature for 1 h. After evaporation, the resulting residue was dissolved in MeCN and reprecipitated with Et_2_O to afford ^**10**^**B-12b** (55.6 mg, 0.113 mmol, 74%) as colorless powder: mp 134–136 °C; ^1^H NMR (400 MHz, D_2_O, TSP): δ 3.03 (t, *J* = 6.0 Hz, 4H), 3.19 (br s, 4H), 3.57 (br s, 4H), 3.95 (s, 2H), 7.51 (t, *J* = 7.6 Hz, 1H), 7.57 (d, *J* = 7.6 Hz, 1H), 7.78–7.80 (m, 2H) ppm; ^13^C NMR (100 MHz, D_2_O, 1,4-dioxane): δ 42.77, 44.17, 48.29, 59.74, 116.92, 129.03, 133.26, 134.00, 135.70, 135.91, 163.61 ppm; IR (ATR) ν: 2808, 1667, 1489, 1439, 1366, 1313, 1180, 1126, 1012, 834, 797, 719, 591, 517, 417 cm^–1^; HRMS (ESI^+^) *m*/*z*: calcd for [M + H]^+^ C_13_H_23_^10^BN_3_O_2_, 263.1914; found, 263.1922; Anal. Calcd (%) for C_13_H_22_^10^BN_3_O_2_·2TFA: C, 41.64; H, 4.93; N, 8.57. Found: C, 41.94; H, 5.02; N, 8.50.

#### 1-[3-(13,15-Dioxa-15-boradispiro[5.0.5.3]pentadec-14-yl)phenyl]methyl-1,4,7-triazacyclononane (^**10**^**B-15b**)

A mixture of ^**10**^**B-12b** (24 mg, 0.049 mmol) and bicyclohexyl-1,1′-diol **26**(35) (10.1 mg, 0.043 mmol, 1.0 equiv) in EtOH (0.7 mL) was refluxed for 13 h. After evaporation, the resulting residue was purified by NH silica gel column chromatography (CHCl_3_/MeOH = 20/1) to afford ^**10**^**B-15b** (18.7 mg, 0.051 mmol, 90%) as a colorless amorphous solid: mp 58–59 °C; ^1^H NMR (400 MHz, CDCl_3_, TMS): δ 1.16–1.33 (m, 6H), 1.71–1.84 (m, 14H), 2.63–2.69 (m, 8H), 2.81 (s, 4H), 3.74 (s, 2H), 7.33 (t, *J* = 7.6 Hz 1H), 7.46 (d, *J* = 7.6 Hz 1H), 7.74 (d, *J* = 7.2 Hz 1H), 7.80 (s, 1H) ppm; ^13^C NMR (100 MHz, CDCl_3_, TMS): δ 22.33, 25.80, 32.45, 46.51, 46.86, 52.95, 61.54, 84.69, 127.73, 131.66, 133.59, 135.22, 138.86 ppm; IR (ATR) ν: 2930, 2854, 1612, 1449, 1397, 1372, 1284, 1242, 1146, 1132, 1091, 1020, 937, 823, 750, 727, 678, 662, 616, 495, 404 cm^–1^; HRMS (ESI^+^) *m*/*z*: calcd for [M + H]^+^ C_25_H_41_^10^BN_3_O_2_, 425.3323; found, 425.3323; Anal. Calcd (%) for C_25_H_40_^10^BN_3_O_2_·0.4CHCl_3_·1.6MeOH: C, 61.93; H, 9.01; N, 8.02. Found: C, 61.66; H, 8.65; N, 7.65.

#### 1-[(3-Boronopheny)methyl]-1,4,7,10-tetraazacyclododecane TFA Salt (2TFA) (^**10**^**B-13b**)^20^

A mixture of 3-(bromomethyl)phenylboronic acid ^**10**^**B-24b**(36) (25.7 mg, 0.12 mmol, 1.1 equiv), 3Boc-cyclen **27**(38) (51.2 mg, 0.108 mmol), and potassium carbonate (18.5 mg, 0.134 mmol, 1.2 equiv) in MeCN (1.5 mL) was refluxed for 10 h. After adding H_2_O, the reaction mixture was extracted with CHCl_3_. The organic layer was washed with brine, dried over Na_2_SO_4_, and concentrated under reduced pressure. The resulting residue was purified by silica gel column chromatography (CHCl_3_/MeOH = 50/1) to afford ^**10**^**B-28b** (83.2 mg) as a colorless amorphous solid.

TFA (1.0 mL) was added to a solution of ^**10**^**B-28b** in CH_2_Cl_2_ (1.0 mL), and the mixture was stirred at room temperature for 1 h. After evaporation, the resulting residue was dissolved in MeCN and reprecipitated with Et_2_O to afford ^**10**^**B-13b** (54.4 mg, 0.102 mmol, 94%) as colorless powder: mp 113–117 °C; ^1^H NMR (400 MHz, D_2_O, TSP): δ 2.93–3.02 (m, 8H), 3.16–3.23 (m, 8H), 3.89 (s, 2H), 7.51–7.53 (m, 2H), 7.75 (s, 1H), 7.79–7.81 (m, 1H) ppm; ^13^C NMR (100 MHz, D_2_O, 1,4-dioxane): δ 42.33, 42.45, 44.75, 48.37, 57.23, 116.91, 129.22, 132.98, 134.07, 135.47, 135.53, 163.60 ppm; IR (ATR) ν: 3018, 2859, 1668, 1404, 1175, 1123, 1064, 830, 796, 718, 629, 593, 517, 411 cm^–1^; HRMS (ESI^+^) *m*/*z*: calcd for [M + H]^+^ C_15_H_28_^10^BN_4_O_2_, 306.2336; found, 306.2334; Anal. Calcd (%) for C_15_H_27_^10^BN_4_O_2_·2.4TFA: C, 41.07; H, 5.12; N, 9.68. Found: C, 40.84; H, 5.32; N, 9.79.

#### 1-[3-(13,15-Dioxa-15-boradispiro[5.0.5.3]pentadec-14-yl)phenyl]methyl-1,4,7,10-tetraazacyclododecane (^**10**^**B-16b**)

A mixture of ^**10**^**B-13b** (26 mg, 0.049 mmol) and bicyclohexyl-1,1′-diol **26**(35) (10.1 mg, 0.051 mmol, 1.0 equiv) in EtOH (1.0 mL) was refluxed for 8 h. After evaporation, the resulting residue was purified by NH silica gel column chromatography (CHCl_3_/MeOH = 20/1) to afford ^**10**^**B-16b** (16.1 mg, 0.034 mmol, 71%) as a colorless amorphous solid: mp 56–58 °C; ^1^H NMR (400 MHz, CDCl_3_, TMS): δ 1.13–1.32 (m, 6H), 1.62–1.80 (m, 14H), 2.56–2.59 (m, 8H), 2.67 (t, *J* = 4.8, 4H), 2.81 (t, *J* = 4.8 Hz, 4H), 3.65 (s, 2H), 7.32 (t, *J* = 8.0 Hz, 1H), 7.40 (d, *J* = 8.0 Hz, 1H), 7.74 (d, *J* = 7.2 Hz, 1H), 7.77 (s, 1H) ppm; ^13^C NMR (100 MHz, CDCl_3_, TMS): δ 22.31, 25.81, 32.44, 45.30, 46.43, 47.37, 51.38, 59.18, 84.57, 127.69, 131.77, 133.77, 135.43, 138.06 ppm; IR (ATR) ν: 2930, 2851, 1579, 1435, 1392, 1371, 1347, 1272, 1243, 1201, 1131, 1076, 1040, 939, 808, 748, 713, 660, 618, 507, 412 cm^–1^; HRMS (ESI^+^) *m*/*z*: calcd for [M + H]^+^ C_27_H_46_^10^BN_4_O_2_, 468.3745; found, 468.3741; Anal. Calcd (%) for C_27_H_45_^10^BN_4_O_2_·0.5CHCl_3_·MeOH: C, 61.19; H, 8.92; N, 10.02. Found: C, 61.27; H, 9.08; N, 9.64.

#### 1-[(4-Boronopheny)methyl]-1,4,7,10,13-pentaazacyclopentadecane TFA Salt (3TFA) (^**10**^**B-14a**)

A mixture of 4-(bromomethyl)phenylboronic acid ^**10**^**B-24a**(34) (38.2 mg, 0.178 mmol, 1.2 equiv), **29**(39) (92.1 mg, 0.150 mmol), and potassium carbonate (25.5 mg, 0.184 mmol, 1.2 equiv) in MeCN (1.5 mL) was refluxed for 11 h. After adding H_2_O, the reaction mixture was extracted with CHCl_3_. The organic layer was washed with brine, dried over Na_2_SO_4_ and concentrated under reduced pressure. The resulting residue was purified by silica gel column chromatography (CHCl_3_/MeOH = 50/1) to afford ^**10**^**B-30a** (112.2 mg) as a colorless amorphous solid.

TFA (1.5 mL) was added to a solution of ^**10**^**B-30a** in CH_2_Cl_2_ (1.5 mL), and the mixture was stirred at room temperature for 1 h. After evaporation, the resulting residue was dissolved in MeCN and reprecipitated with Et_2_O to afford ^**10**^**B-14a** (79.1 mg, 0.115 mmol, 77%) as colorless powder: mp 136–140 °C; ^1^H NMR (400 MHz, D_2_O, TSP): δ 2.99 (t, *J* = 5.2 Hz, 4H), 3.16 (s, 4H), 3.24–3.33 (m, 12H), 3.95 (s, 2H), 7.45 (d, *J* = 8.0 Hz, 2H), 7.84 (d, *J* = 7.6 Hz, 2H) ppm; ^13^C NMR (100 MHz, D_2_O, 1,4-dioxane): δ 44.29, 45.29, 45.78, 50.21, 56.67, 116.90, 130.55, 134.65, 137.01, 163.59 ppm; IR (ATR) ν: 3045, 2852, 1671, 1610, 1421, 1391, 1200, 1177, 1126, 1060, 1016, 836, 799, 735, 720, 699, 666, 518, 503, 413 cm^–1^; HRMS (ESI^+^) *m*/*z*: calcd for [M + H]^+^ C_17_H_33_^10^BN_5_O_2_, 349.2758; found, 349.2754; Anal. Calcd (%) for C_17_H_32_^10^BN_5_O_2_·3TFA: C, 40.00; H, 5.11; N, 10.14. Found: C, 40.13; H, 5.03; N, 10.07.

#### 1-[4-(13,15-Dioxa-15-boradispiro[5.0.5.3]pentadec-14-yl)phenyl]methyl-1,4,7,10,13-pentaazacyclopentadecane (^**10**^**B-17a**)

A mixture of ^**10**^**B-14a** (32.5 mg, 0.047 mmol) and bicyclohexyl-1,1′-diol **26**(35) (9.7 mg, 0.049 mmol, 1.0 equiv) in EtOH (0.7 mL) was refluxed for 7 h. After evaporation, the resulting residue was purified by NH silica gel column chromatography (CHCl_3_/MeOH = 20/1) to afford ^**10**^**B-17a** (20.9 mg, 0.041 mmol, 87%) as a colorless amorphous solid: mp 47–49 °C; ^1^H NMR (400 MHz, CDCl_3_, TMS): δ 1.16–1.32 (m, 6H), 1.62–1.83 (m, 14H), 2.64 (s, 12H), 2.78 (s, 8H), 3.61 (s, 2H), 7.33 (d, *J* = 7.6 Hz, 2H), 7.80 (d, *J* = 8.0 Hz, 2H) ppm; ^13^C NMR (100 MHz, CDCl_3_, TMS): δ 22.29, 25.80, 32.45, 47.34, 47.91, 48.41, 49.14, 54.66, 59.54, 84.61, 128.30, 134.90, 142.52 ppm; IR (ATR) ν: 2929, 2849, 1612, 1517, 1448, 1396, 1373, 1243, 1131, 1091, 1040, 1020, 937, 823, 747, 730, 677, 661, 507 cm^–1^; HRMS (ESI^+^) *m*/*z*: calcd for [M + H]^+^ C_29_H_51_^10^BN_5_O_2_, 511.4167; found, 511.4166; Anal. Calcd (%) for C_29_H_50_^10^BN_5_O_2_·0.3CHCl_3_·2MeOH: C, 61.56; H, 9.62; N, 11.47. Found: C, 61.77; H, 9.25; N, 11.15.

### X-ray Data Collection and Refinement

The crystals of **19a** were suitable for a single-crystal X-ray structure analysis, which were performed on a Bruker APEX CCD diffractometer equipped with a Rigaku Instruments low-temperature attachment. Data were collected at 93 K using monochromated Mo Kα radiation (λ = 0.71073 Å). The frames were indexed, integrated, and scaled using the SMART and SAINT software packages. An empirical absorption correction was applied to the collection reflections with SADABS using XPREP. The structure was solved by the direct method and refined on *F*_2_ by the full-matrix least squares technique using the SHELX-2015 program package. All non-hydrogen atoms were refined anisotropically. The crystal data in this manuscript can be obtained free of change from The Cambridge Crystallographic Data Centre via www.cccdc.cam.ac.uk/data_request/cif. Crystal data for **19a** C_29_H_51_BN_6_O_9_Zn, *M*_r_ = 703.93, orthorhombic, *P* 2_1_ 2_1_ 2_1_, *a* = 10.008 (4), *b* = 10.967 (4), *c* = 30.483 (12) Å, *V* = 3346 (2) Å^3^, *Z* = 4, ρ_calc_ = 1.397 g·cm^–3^, *R* = 0.0553 (7083 reflections), *R*_w_ = 0.1229 (7656 reflections), GOF = 1.195. CCDC 2058200 contains the supplementary crystallographic data for the paper.

### Cell Cultures

HeLa S3 cells (human cervical carcinoma) were cultured in Minimum essential medium (MEM) containing 10% FBS, penicillin, and streptomycin. A549 cells (human caucasian lung carcinoma) and IMR-90 cells (normal human fibroblast) were cultured in Dulbecco’s modified Eagle’s medium (DMEM) with 10% FBS, penicillin, and streptomycin. All cells were cultured at 37 °C in a humidified atmosphere containing 5% CO_2_.

### MTT Assays

HeLa S3, A549, and IMR-90 cells (1 × 10^4^ cells/well) were seeded on 96-well plates (Watson) in cell culture medium. After incubation overnight at 37 °C under 5% CO_2._, the cells were treated with ^10^B-BSH **1** (Stella Chemifa, Japan, ^10^B-enrichment ≥ 95%), BPA **2** (Fluka, USA)-d-fructose complex, **7** and **12**–**20** (0–200 μM) in cell culture medium under same conditions for 24 h, and then, 0.5% MTT reagent in PBS (10 μL) was added to each well. After incubation for 4 h, a formazan lysis solution (10% sodium dodecyl sulfate in 0.01 N HCl aq) (100 μL) was added and the resulting solution was incubated under same conditions overnight. The absorbance at λ = 570 nm was measured with a microplate reader (Bio-Rad).

### Measurement of Intracellular Uptake of Boron Compounds into HeLa S3, A549, and IMR-90 Cells Evaluated by ICP–MS

HeLa S3, A549, and IMR-90 cells (5 × 10^5^ cells/well) were seeded on 6-well plates (TrueLine, USA) in cell culture medium. After incubation overnight at 37 °C under 5% CO_2_ and 18–20% O_2_ (normoxic conditions), the cells were washed gently with PBS (1 mL) and treated with the boron compounds **1**, **2**, **7**, and **12**–**20** (30 μM) in cell culture medium (2 mL) under same conditions for 24 h (*n* = 4). To count the number of cells after treatment with the boron compounds, the cells (*n* = 1) were washed with PBS, detached by trypsin, and counted with a hemocytometer. For measurement of boron uptake, the cells (*n* = 3) were washed with PBS (1 mL ×3) and digested with 60% HNO_3_ aq (0.5 mL) at room temperature for 24 h, which were transferred to 15 mL centrifuge tubes with Milli-Q water (3.5 mL). These tubes were centrifuged at 3000 rpm and 4 °C for 10 min, and the resulting sample solutions were filtered. The concentration of boron atoms was determined by ICP–MS (NexION300S, PerkinElmer, Waltham, Massachusetts, USA).

### Active Energy-Dependent Uptake of Boron-Containing Macrocyclic Polyamine Derivatives into HeLa S3 and A549 Cells

HeLa S3 and A549 cells (5 × 10^5^ cells/well) were seeded on 6-well plates (TrueLine, USA) and incubated in cell culture medium at 37 °C under 5% CO_2_ (*n* = 4). After incubation for 2 days, the cells were washed with PBS (1 mL) and treated with boron compounds **2** and **15a**–**17a** (30 μM) in cell culture medium (2 mL) at 37 °C or 4 °C for 1 h (*n* = 4). To count the number of cells after treatment with the boron compounds, the cells (*n* = 1) were washed with PBS, detached by trypsin, and counted with a hemocytometer. For measurement of boron uptake, the cells (*n* = 3) were washed with PBS (1 mL ×3) and digested with 60% HNO_3_ aq (0.5 mL) at room temperature for 24 h and then transferred to 15 mL centrifuge tubes with Milli-Q water (3.5 mL). These tubes were centrifuged at 3000 rpm and 4 °C for 10 min and then sample solution was filtered. The concentration of boron atoms was determined by ICP–MS (NexION300S, PerkinElmer, Waltham, Massachusetts, USA).

### Effect of Inhibitors on the Intracellular Uptake of **17a**

HeLa S3 and A549 cells (5 × 10^5^ cells/well) were seeded on 6-well plates (TrueLine, USA) and incubated in cell culture medium at 37 °C under 5% CO_2_ for 2 days (*n* = 4). After preincubation with inhibitors in cell culture medium (2 mL) at 37 °C for 1 h, the cells were treated with boron compounds **2** and **17a** (30 μM) in the presence of inhibitors at 37 °C for 1 h. To count the number of cells after treatment with the boron compounds, the cells (*n* = 1) were washed with PBS, detached by trypsin, and counted with a hemocytometer. For measurement of boron uptake, the cells (*n* = 3) were washed with PBS (1 mL ×3) and digested with 60% HNO_3_ aq (0.5 mL) at room temperature for 24 h, which were transferred to 15 mL centrifuge tubes with Milli-Q water (3.5 mL). These tubes were centrifuged at 3000 rpm and 4 °C for 10 min and then sample solutions were filtered. The concentration of boron atoms was determined by ICP–MS (NexION300S, PerkinElmer, Waltham, Massachusetts, USA).

### Evaluation of the Anti-tumor Effect of Boron-Containing Macrocyclic Polyamine Derivatives with Thermal Neutron Irradiation (Colony Formation Assay)

A549 cells (5 × 10^5^ cells/well) were seeded on 6-well plates (TrueLine, USA) and incubated in cell culture medium at 37 °C under 5% CO_2_ for 1 day. After removing the cell culture medium, the cells were washed gently with PBS. Cell culture medium containing 30 μM of boron compounds (2 mL) was added to the wells, which was incubated for 24 h under same conditions. After removing the medium, the cells were washed twice with PBS (1.0 mL) and collected by trypsinization. After centrifugation, the supernatant was removed, and cell culture medium was added to prepare a cell suspension (5 × 10^4^ cells/mL). The cells (5 × 10^4^ cells/mL, 1 mL) in 1.5 mL tubes were irradiated with thermal neutrons (Institute for Integrated Radiation and Nuclear Science, Kyoto University, Osaka, Japan) for 0, 15, 30, and 45 min, respectively. The thermal neutron flux (1.5 × 10^9^ n/cm^2^·s) was measured by two gold foils which were attached to the surface of the 1.5 mL tube. To evaluate the cell proliferation, the irradiated cells (3 × 10^3^ cells/well, 1.0 mL) were seeded on 12-well plates (TrueLine, USA) and incubated for 7 days at 37 °C under 5% CO_2_ in cell culture medium. After removing the medium, the attached cells were washed gently with PBS, fixed with EtOH, stained by 0.1% crystal violet, and washed with PBS three times.

For analyzing the cell proliferation, images of the stained colony were acquired using a Bio-Rad Chemidoc MP Imaging System (Bio-Rad, Hercules, CA, USA), which were automatically examined by ImageJ-plugin Colony Area to determine the percentage of colony area of each wells.^43^ The surviving fractions were calculated as the colony area and normalized by the result for non-irradiated condition.

### Effect of Boron-Containing Macrocyclic Polyamine Derivatives on the Melting Temperature of ctDNA

Thermal denaturation experiments of ctDNA (50 μM in phosphate) in 10 mM HEPES buffer (pH 7.4) with *I* = 0.02 (NaNO_3_) were performed on a JASCO V-550 UV/vis spectrophotometer (JASCO, Tokyo, Japan) equipped with a thermoelectric temperature controller (±0.5 °C), a stirring unit, and a 10 mm quartz cuvette. All aqueous solutions were made with purified water. The concentration of ctDNA was determined by UV absorption spectroscopy based on its molar extinction coefficient at 253 nm (ε_253_ = 6.6 × 10^3^).^31d,45^ Thermal melting curves for ctDNA with and without additives (**16b**, **19b**, **17a**, **20a**, and **3**) were obtained by following the absorption change at 260 nm as a function of the temperature (the temperature was raised at the rate of 1 °C/min). The *T*_m_ value was graphically determined from the spectral data, and the Δ*T*_m_ value for each condition was calculated from the results in the presence and absence of additives.

## Supporting Information Available

Structures of endocytosis inhibitors (MβCD, dynasore, and amiloride), results of MTT assay of **1**, **2**, **7**, and **12**–**20** against HeLa S3, A549, and IMR-90 cells, intracellular uptake of **2** and **17a** into A549 cells in the presence and absence of spermidine (**3**), images of colony formation assay of A549 cells after thermal neutron irradiation, and results of thermal denaturation experiments of ctDNA (PDF)
Crystallographic data of boron uptake of HeLa S3, A549, and IMR-90 cells (CSV)

## Abbreviations

ATB^0,+^: amino acid transporter system B^0,+^
ATR: attenuated total reflection
A549 cells: human caucasian lung carcinoma
brs: broad signal
BNCT: boron neutron capture therapy
BPA: l-4-boronophenylalanine
BPO: benzoyl peroxide
BSH: sodium mercaptoborate
*n*-BuLi: *n*-butyllithium
ctDNA: calf thymus DNA
dT: thymidine
DMEM: Dulbecco’s modified Eagle medium
FBS: fetal bovine serum
GLUT: glucose transporter
HeLa S3 cells: human cervical carcinoma
IMR-90 cells: normal human fibroblast
ICP–MS: inductively coupled plasma mass spectrometry
Jurkat cells: human T lymphocyte cells
KURNS: The Institute for Integrated Radiation and Nuclear Science, Kyoto University
LAT: L-type amino acid transporter
LET: linear energy transfer
MTT: 3-(4,5-dimethylthiazol-2yl)-2,5-diphenyltetrazolium bromide
MβCD: methyl-beta-cyclodextrin
MEM: minimum essential medium
PTS: polyamine transport system
quant: quantitative
SD: standard deviation
SQAG: sulfoquinovosyl acyl glycerol
T/B: tumor to blood
T/N: tumor to normal cell
THF: tetrahydrofuran
TSP: 3-(trimethylsilyl)propionic-2,2,3,3-*d*_4_ acid sodium
[9]aneN_3_: 1,4,7-triazacyclononane
[12]aneN_4_: 1,4,7,10-tetraazacyclododecane (cyclen)
[15]aneN_5_: 1,4,7,10,13-pentaazacyclopentadecane
