# Supplementary material for: Design, Synthesis, and Biological Evaluation of Boron-Containing Macrocyclic Polyamines and Their Zinc(II) Complexes for Boron Neutron Capture Therapy
Source: J Med Chem. 2021 Jun 2;64(12):8523–44. doi: 10.1021/acs.jmedchem.1c00445 (PMC8279495; doi:10.1021/acs.jmedchem.1c00445)
Supplement: Supplementary file 1 — jm1c00445_si_001.pdf [file jm1c00445_si_001.pdf]

Supplementary Data

Design, Synthesis and Biological Evaluation of Boron-Containing Macrocyclic Polyamines and Their Zinc(II) Complexes for Boron Neutron Capture Therapy (BNCT)

Hiroki Ueda,[a] *Minoru Suzuki,*[b] *Reiko Kuroda,*[c]
Tomohiro Tanaka,[a] *and Shin Aoki* *[,a,c,d]

[a] *Faculty of Pharmaceutical Sciences, Tokyo University of Science, 2641 Yamazaki, Noda, Chiba 278-8510, Japan*
[b] *Institute for Integrated Radiation and Nuclear Science, Kyoto University, 2-Asashiro-nishi, Kumatori, Osaka 590-0494, Japan*
[c] *Research Institute for Science and Technology, Tokyo University of Science, 2641 Yamazaki, Noda, Chiba 278-8510, Japan*
[d] *Research Institute for Biomedical Sciences, Tokyo University of Science, 2641 Yamazaki, Noda, Chiba 278-8510, Japan*

*Corresponding authors: E-mail, shinaoki@rs.tus.ac.jp (Shin Aoki)

1. **Structures of Endocytosis Inhibitors** S3

2. **MTT Assay with Boron Compounds against HeLa S3, A549 and IMR-90 Cells**

Figure S1. Result of HeLa S3 cells S4

Figure S2. Result of A549 cells S5

Figure S3. Result of IMR-90 cells S6

3. **Effect of Spermidine on the Intracellular Uptake of 2 and 17a into A549 Cells** S7

4. **Results of Colony Formation Assay of A549 Cells after BNCT with Boron-containing Compounds (Images)** S8

5. **Results of Thermal Denaturation Experiments of Calf Thymus DNA in the Presence of 3, 16b, 19b, 17a and 20a**

Figure S6. Change in thermal melting (T_m) curves of ctDNA S9

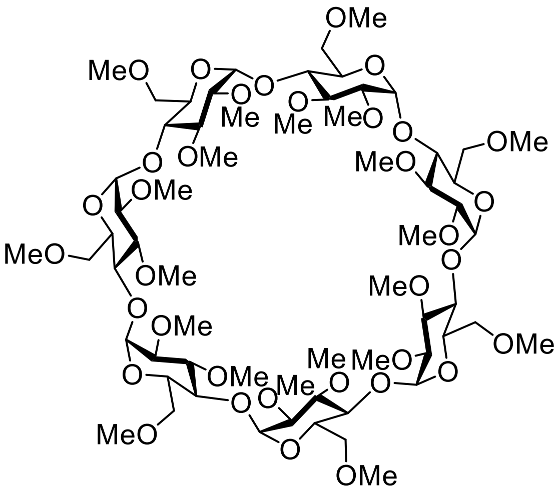

Methyl-β-cyclodextrin (MβCD)

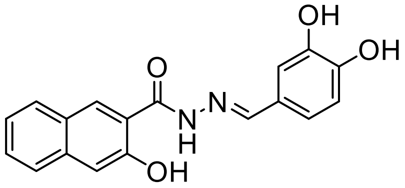

Dynasore

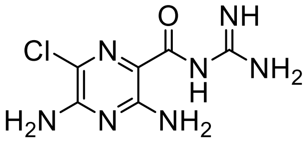

Amiloride

Scheme S1. Structures of endocytosis inhibitors

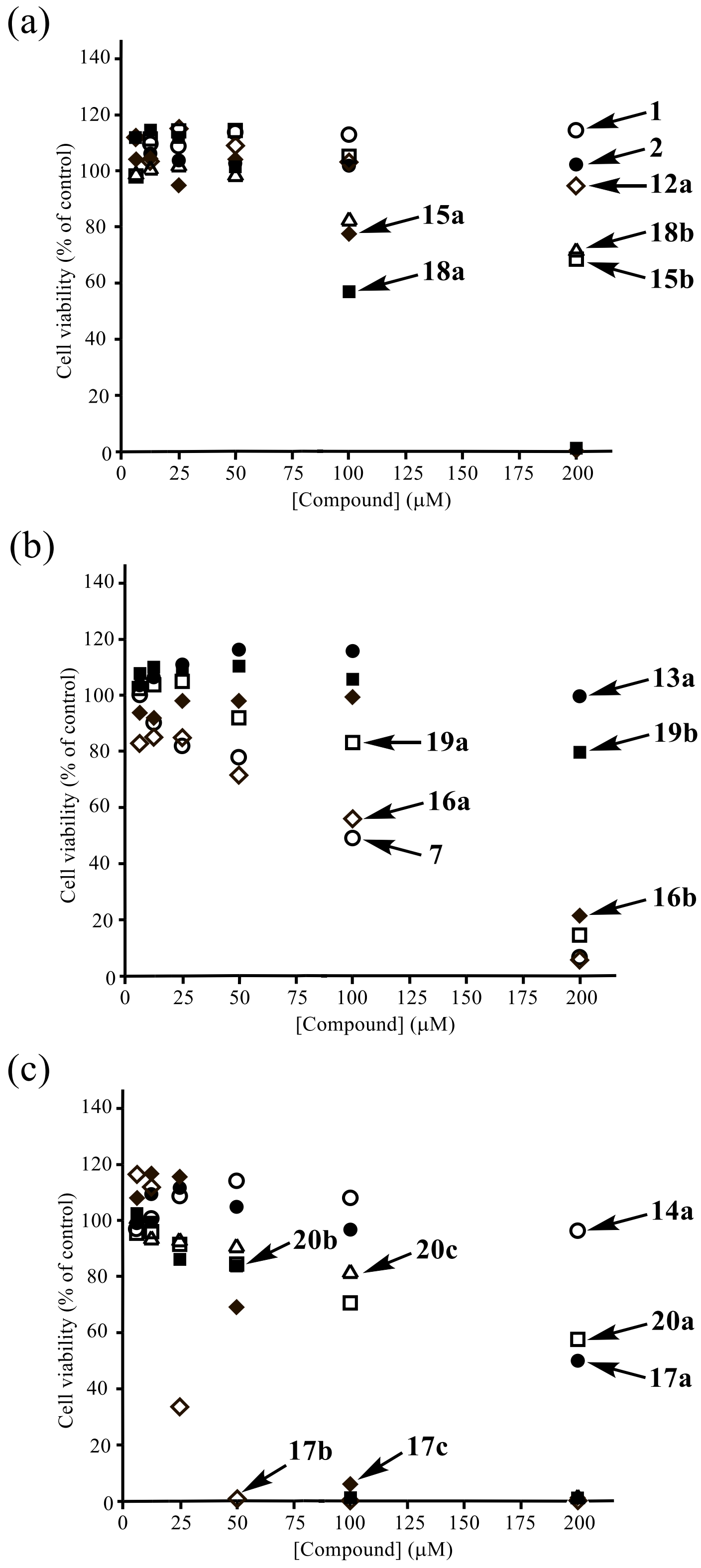

Figure S1. Results of MTT assay with boron compounds **1**, **2**, **7** and **12**–**20** against HeLa S3 cells. Cell viability of HeLa S3 cells (% of control; in the absence of boron compound) in the presence of boron compounds **1** (○), **2** (●), **12a** (◇), **15a** (◆), **15b** (□), **18a** (■) and **18b** (△) (a), **7** (○), **13a** (●), **16a** (◇), **16b** (◆), **19a** (□) and **19b** (■) (b), **14a** (○), **17a** (●), **17b** (◇), **17c** (◆), **20a** (□), **20b** (■) and **20c** (△) (c) [0–200 μM] at 37 °C for 24 h.

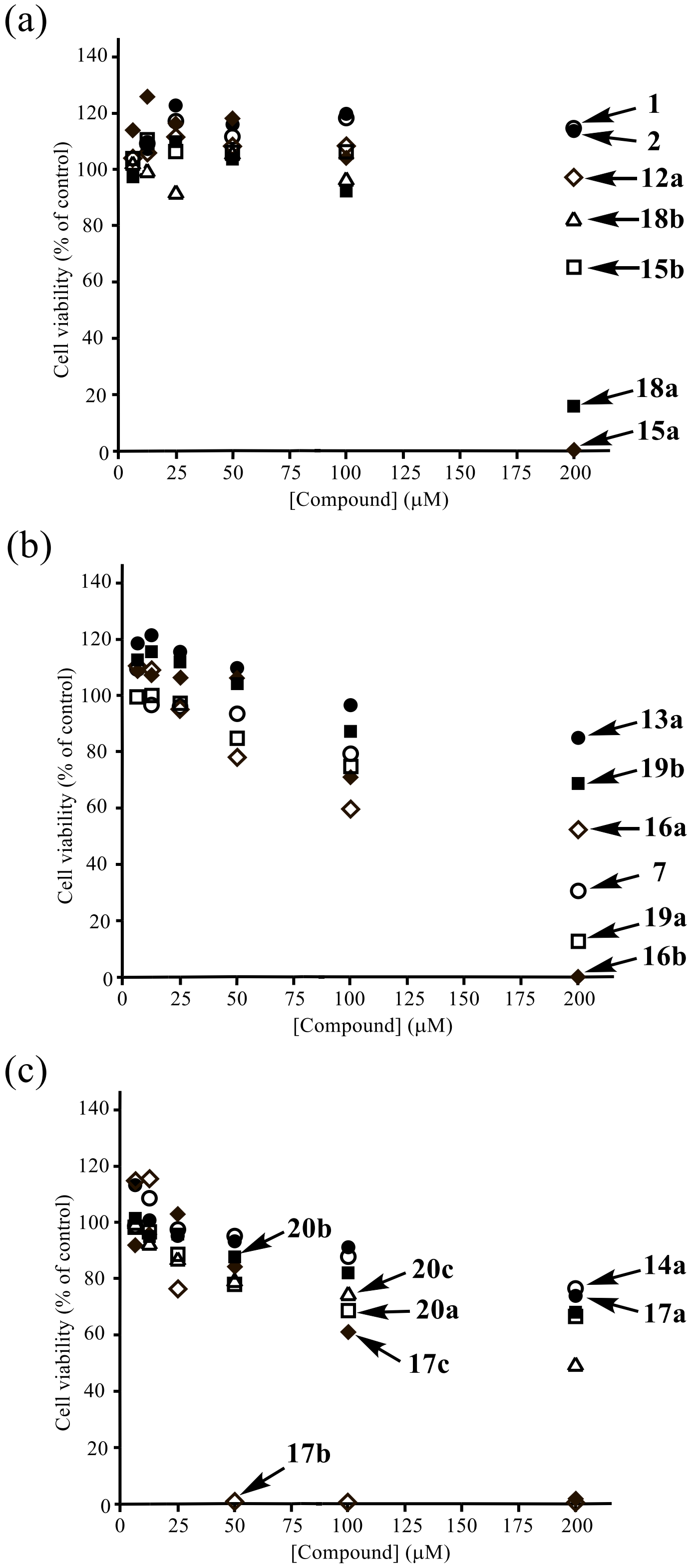

Figure S2. Results of MTT assay with boron compounds **1**, **2**, **7** and **12–20** against A549 cells. Cell viability of A549 cells (% of control; in the absence of boron compound) in the presence of boron compounds **1** (○), **2** (●), **12a** (◇), **15a** (◆), **15b** (□), **18a** (■) and **18b** (△) (a), **7** (○), **13a** (●), **16a** (◇), **16b** (◆), **19a** (□) and **19b** (■) (b), **14a** (○), **17a** (●), **17b** (◇), **17c** (◆), **20a** (□), **20b** (■) and **20c** (△) (c) [0–200 μM] at 37 °C for 24 h.

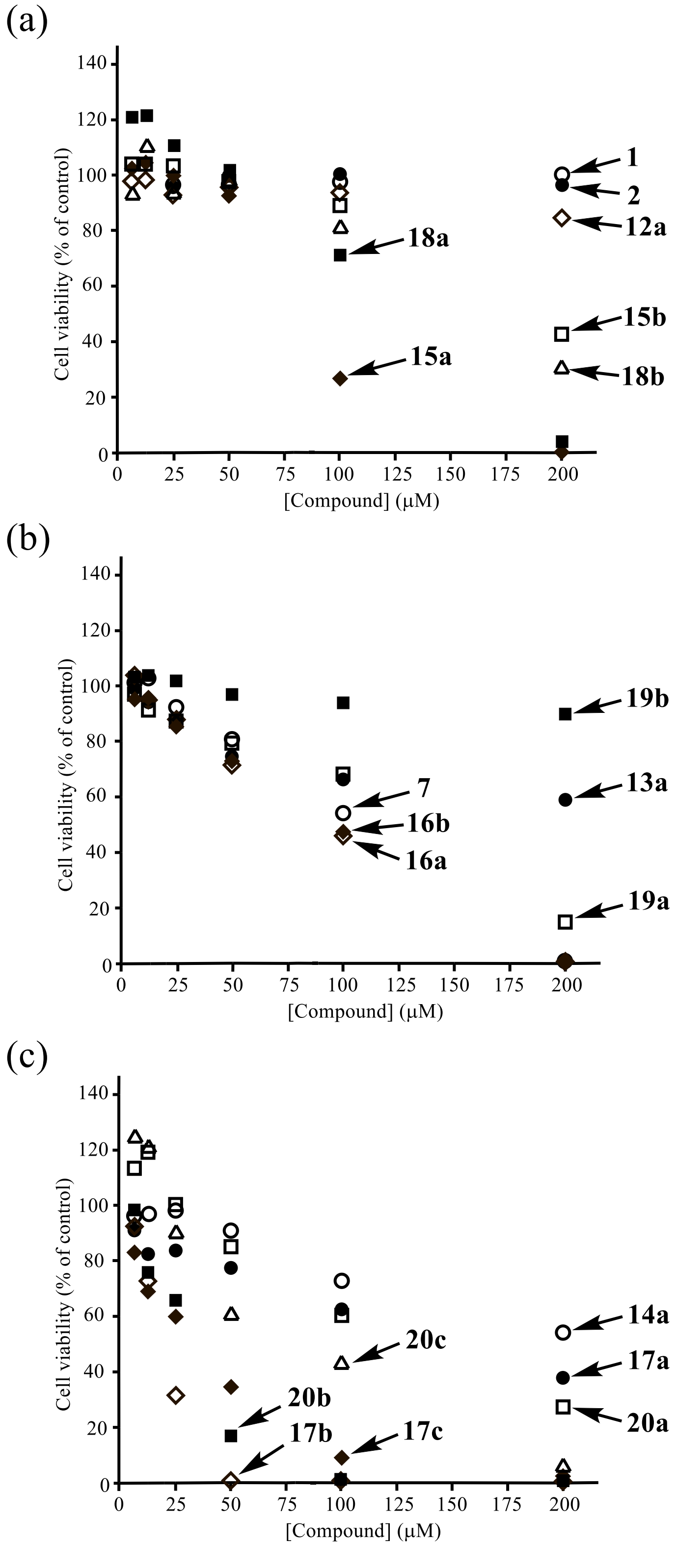

Figure S3. Results of MTT assay with boron compounds **1**, **2**, **7** and **12**–**20** against IMR-90 cells. Cell viability of IMR-90 cells (% of control; in the absence of boron compound) in the presence of boron compounds **1** (○), **2** (●), **12a** (◇), **15a** (◆), **15b** (□), **18a** (■) and **18b** (△) (a), **7** (○), **13a** (●), **16a** (◇), **16b** (◆), **19a** (□) and **19b** (■) (b), **14a** (○), **17a** (●), **17b** (◇), **17c** (◆), **20a** (□), **20b** (■) and **20c** (△) (c) [0–200 μM] at 37 °C for 24 h.

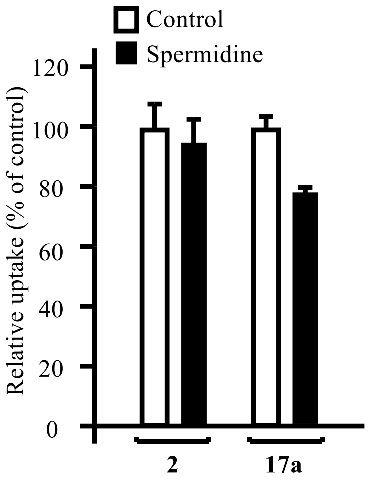

Figure S4. Relative uptake of **2** and **17a** (30 μM) into A549 cells in the absence (open bars) and presence of **3** (2 mM) (closed bars). After preincubation with **3** for 1 h, the cells were incubated with **2** and **17a** at 37 °C for 1 h in the presence of **3**. Data represent the mean ± SD of at least three replicates.

4. Results of Colony Formation Assay of A549 Cells after BNCT with Boron-containing Compounds (Images)

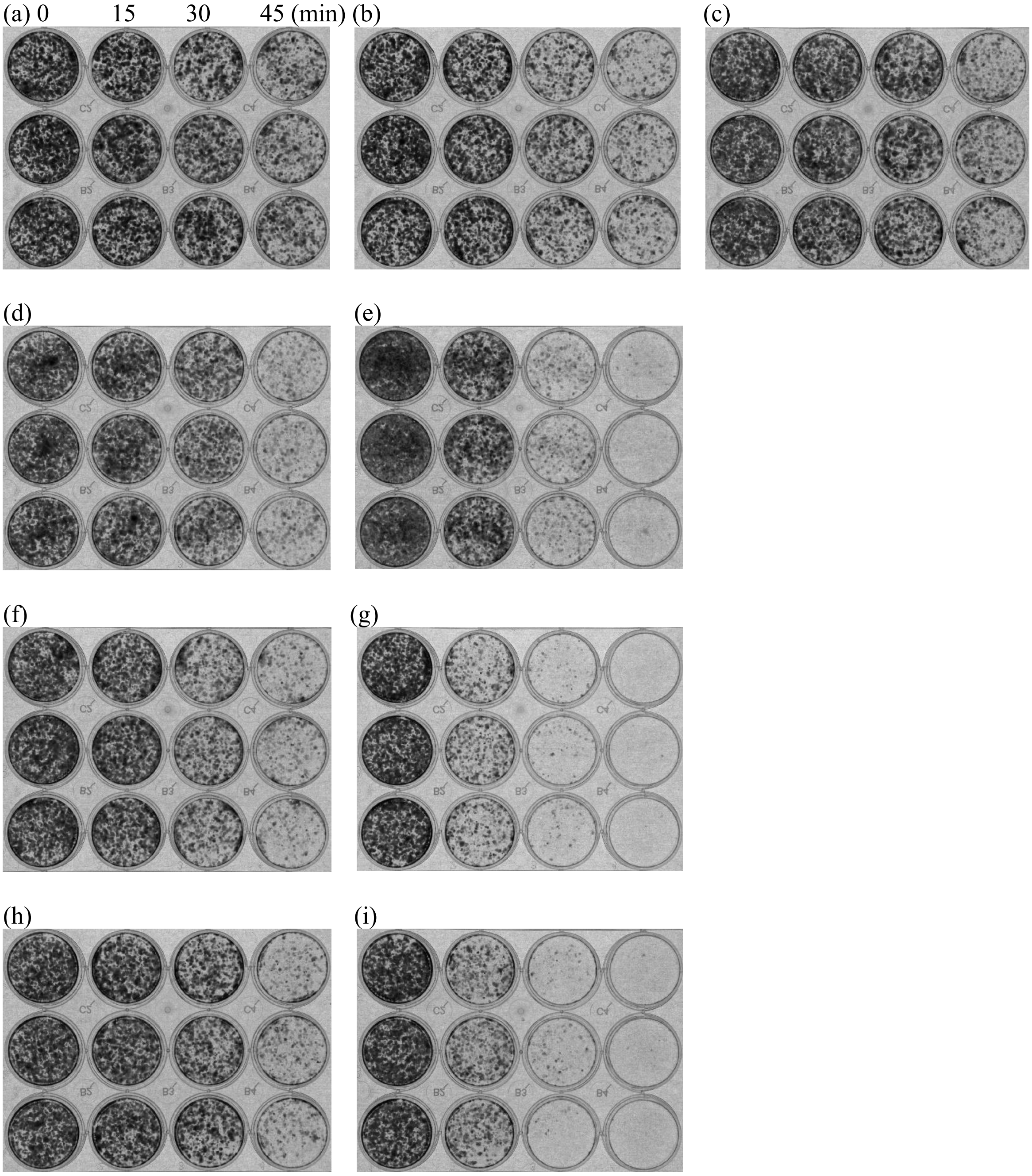

Figure S5. Typical images of colony formation assay of A549 cells after thermal neutron irradiation in the presence of (a) none, (b) ^{10}B-BSH (**1**), (c) ^{10}B-BPA (**2**), (d) **15b**, (e) **^{10}B-15b**, (f) **16b**, (g) **^{10}B-16b**, (h) **17a**, and (i) **^{10}B-17a** ([B-containing compounds] = 30 μM) (taken by Bio-Rad ChemiDoc^{TM} MP Imaging System (Bio-Rad)).

5. Results of Thermal Denaturation Experiments of Calf Thymus DNA

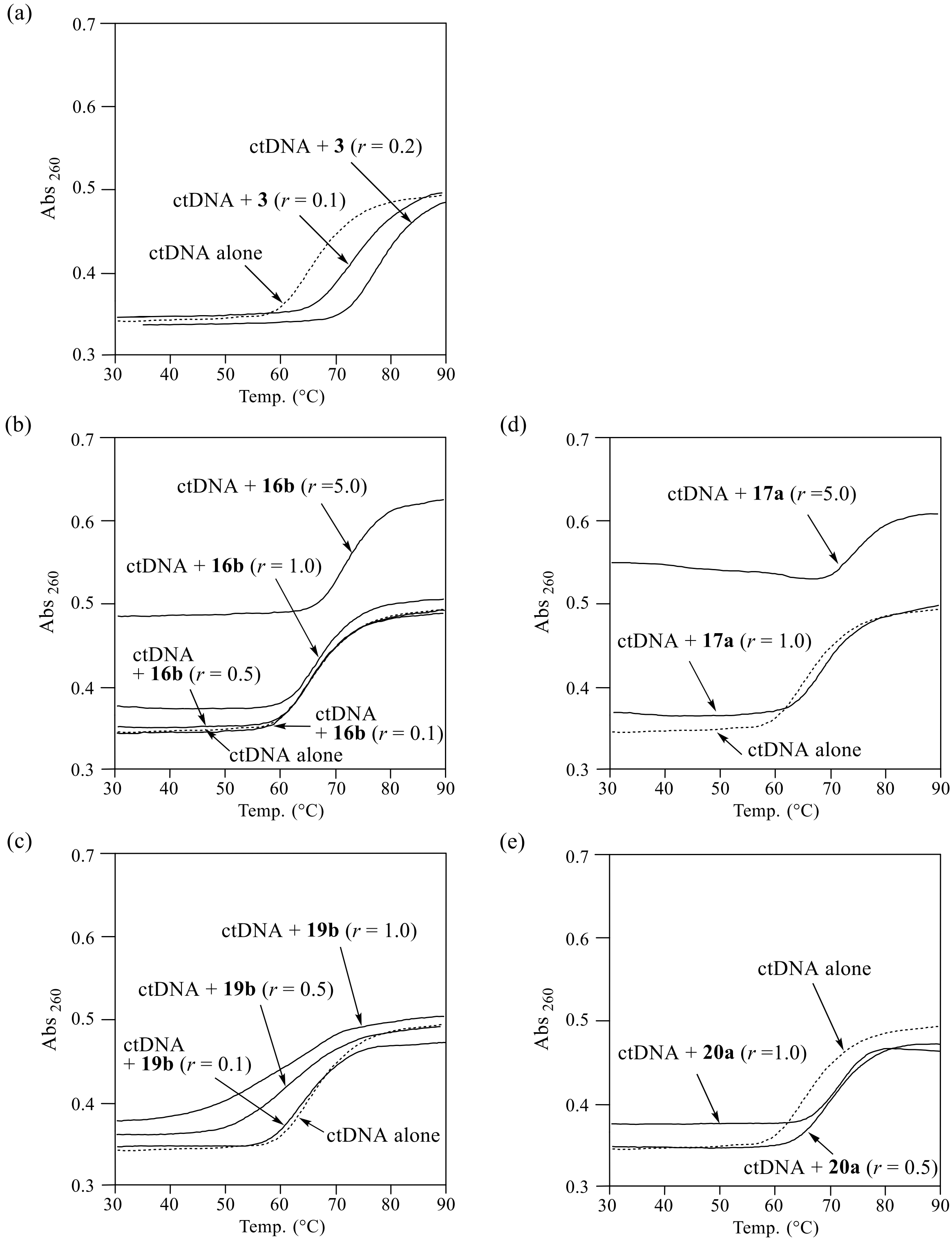

Figure S6. Change in thermal melting (T_m) curves of ctDNA (at [ctDNA(P)] = 50 μM) in the absence (dash line) and presence of (a) **3**, (b) **16b**, (c) **19b**, (d) **17a** and (e) **20a** (plain line) at pH 7.4 (10 mM HEPES with I = 0.02 ($NaNO_3$), (r = [**3**, **16b**, **19b**,**17a** or **20a**]/[ctDNA(P)])
